# Coordinated repression and activation of two transcriptional programs stabilizes cell fate during myogenesis

**DOI:** 10.1242/dev.101956

**Published:** 2014-07

**Authors:** Lucia Ciglar, Charles Girardot, Bartek Wilczyński, Martina Braun, Eileen E. M. Furlong

**Affiliations:** European Molecular Biology Laboratory, Genome Biology Unit, Heidelberg 69117, Germany

**Keywords:** Tramtrack69, Myoblast cell fate specification, Transcriptional networks, Transcriptional repression, Enhancer, *Drosophila*

## Abstract

Molecular models of cell fate specification typically focus on the activation of specific lineage programs. However, the concurrent repression of unwanted transcriptional networks is also essential to stabilize certain cellular identities, as shown in a number of diverse systems and phyla. Here, we demonstrate that this dual requirement also holds true in the context of *Drosophila* myogenesis. By integrating genetics and genomics, we identified a new role for the pleiotropic transcriptional repressor Tramtrack69 in myoblast specification. *Drosophila* muscles are formed through the fusion of two discrete cell types: founder cells (FCs) and fusion-competent myoblasts (FCMs). When *tramtrack69* is removed, FCMs appear to adopt an alternative muscle FC-like fate. Conversely, ectopic expression of this repressor phenocopies muscle defects seen in loss-of-function *lame duck* mutants*,* a transcription factor specific to FCMs. This occurs through Tramtrack69-mediated repression in FCMs, whereas Lame duck activates a largely distinct transcriptional program in the same cells. Lineage-specific factors are therefore not sufficient to maintain FCM identity. Instead, their identity appears more plastic, requiring the combination of instructive repressive and activating programs to stabilize cell fate.

## INTRODUCTION

Diversity in cell fates is generated by multiple mechanisms during development, including asymmetric cell division, and through inductive cues from surrounding tissues. In situations in which immediate neighbors give rise to distinct cell fates, inductive signals alone are often insufficient, as juxtaposed cells may receive similar signaling cues. In these contexts, a cell typically acts to actively repress neighboring cell types from acquiring the same identity. This can be achieved, for example, through Notch-mediated lateral inhibition occurring in part through negative-feedback loops (Heitzler and Simpson, 1991), or through reciprocal inhibition, whereby two key cell identity genes actively repress the expression of each other in neighboring cell types (Jagla et al., 2002; Lagha et al., 2009). In both cases, the fate of a cell is positively regulated through the activation of a specific transcriptional program, while that same program is actively repressed in neighboring cells.

To stabilize cell identity, a cell may therefore need to activate its specific lineage program, while simultaneously repressing the program of cells with a shared developmental history. This has been observed in a number of diverse systems, including *Drosophila* socket cells (Miller et al., 2009), mouse erythroid progenitors (Wontakal et al., 2012) and zebrafish cardiomyocytes (Simoes et al., 2011), and we show here that this dual requirement is also essential for *Drosophila* myogenesis.

The *Drosophila* somatic muscle, which is in many respects analogous to the vertebrate skeletal musculature, consists of a highly organized pattern of 30 distinct multinucleated muscle fibers in each abdominal hemisegment (Bate, 1990). Each fiber is assembled from a single founder cell (FC) and from multiple fusion-competent myoblasts (FCMs) during the process of myoblast fusion [reviewed by Rochlin et al. (2010)]. Initially, the two cell types are derived from an equivalence group through the interplay of Ras, activated by inductive signaling pathways and promoting FC fate, and Notch-mediated lateral inhibition repressing FC fate in the neighboring myoblasts, thereby promoting FCM fate (Carmena et al., 2002; Artero et al., 2003). FCs express a diverse repertoire of transcription factors (TFs) responsible for the unique properties of each muscle fiber [reviewed by Tixier et al. (2010)]. FCMs, by contrast, contribute to the size of all 30 muscles (Bataillé et al., 2010), and yet this myoblast population appears to have both diversity (Rochlin et al., 2010) and plasticity (Sellin et al., 2009). All FCMs express Lame duck (Lmd), a zinc-finger TF, the expression of which initiates in the somatic mesoderm at stage 11, with little or no expression in FCs (Duan et al., 2001; Furlong et al., 2001; Ruiz-Gomez et al., 2002). Lmd is essential for many steps of muscle development, which is reflected by its extensive transcriptional program (Busser et al., 2012): in the dorsal mesoderm it is required for the distinction between FCM and pericardial cell fates (Sellin et al., 2009) and in the visceral mesoderm it is essential for FCM cell identity, while the active exclusion of Lmd protein from FC nuclei is essential for FC cell fate (Popichenko et al., 2013). By directly activating *Myocyte enhancer factor 2* (*Mef2*) (Duan et al., 2001) and *sticks and stones* (*sns*) expression (Ruiz-Gomez et al., 2002; Cunha et al., 2010), Lmd is also required for myoblast fusion. By modulating Mef2 activity within FCMs, it can also elicit diverse transcriptional regulation of genes essential for other aspects of muscle differentiation (Cunha et al., 2010).

In this study, we identify a new role for the TF Tramtrack (Ttk) in the specification of FCM cell fate together with Lmd. Ttk is a zinc-finger repressor found in two isoforms, Ttk69 and Ttk88, which differ in their DNA-binding domains (Read and Manley, 1992). The Ttk88 isoform is not essential for embryogenesis (Xiong and Montell, 1993), but is required for sensory organ (Badenhorst et al., 2002) and photoreceptor (Xiong and Montell, 1993; Lai et al., 1996) development. By contrast, Ttk69 was originally identified as a maternally provided repressor of pair-rule genes in the early blastoderm (Harrison and Travers, 1990; Brown and Wu, 1993). Zygotically expressed Ttk69 plays a crucial role as a repressor of neuronal cell fate in glial cells and other support cells in the central and peripheral nervous system (Giesen et al., 1997; Badenhorst et al., 2002). In addition, Ttk69 is involved in multiple steps of tracheal development (Araujo et al., 2007; Rotstein et al., 2011) and photoreceptor cell fate specification during larval stages (Xiong and Montell, 1993).

Despite these diverse roles in development, no known role for Tramtrack in mesoderm or muscle development has been described to date. In this study, we combine genetics and genome-wide TF occupancy to dissect the contribution of Ttk69 to myogenesis and to understand the molecular mechanisms by which the identified phenotypes arise. We uncover a complex interplay between Ttk69-mediated transcriptional repression of FC genes and Lmd-mediated activation of FCM genes that is required to provide FCMs with their correct cell identity.

## RESULTS

### Tramtrack is dynamically expressed in the mesoderm

**Fig. 1.**
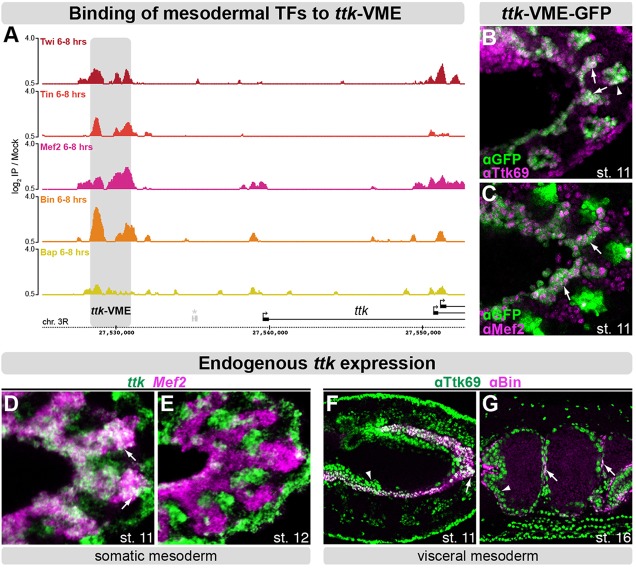
**Tramtrack69 is expressed in the somatic and visceral mesoderm of *Drosophila* embryos.** (A) Binding of five mesodermal TFs in the *ttk* locus at 6-8 h after egg laying [data from Zinzen et al. (2009)]. Genes are depicted at the bottom, the *ttk*-VME CRM is in gray, the asterisk marks a recently annotated gene (see http://flybase.org/reports/FBrf0204466.html). (B,C) Immunostaining against the GFP reporter driven by the *ttk*-VME enhancer (green). (B) CRM activity overlaps expression of endogenous Ttk69 protein (magenta) in mesoderm (arrows) and in tracheal placodes (arrowhead). (C) *ttk*-VME is active in the visceral mesoderm (VM) and in a subset of the somatic mesoderm (SM) (arrows), overlapping with expression of Mef2 protein (magenta). (D,E) Fluorescent *in situ* hybridization against endogenous *ttk* transcripts (green) and the mesodermal marker *Mef2* (magenta). (D) In SM (arrows), *ttk* is expressed transiently at stage 11. (E) At stage 12, there is no detectable *ttk* expression in SM. (F,G) Double immunostaining using a Ttk69-specific antibody (green) and anti-Bin (magenta), marking the VM. (F) At stage 11, Ttk69 is robustly expressed in VM, where it colocalizes with Bin (arrow), and in endoderm (arrowhead). (G) At stage 16, Ttk69 is expressed in both VM (arrows) and endoderm (arrowhead).

Although transcriptional repression is just as important as activation during development (Cook et al., 2003; Atkey et al., 2006; Watson et al., 2011), our understanding of its contribution to myoblast cell fate specification remains limited. We therefore searched for novel transcriptional repressors among putative target genes of essential mesoderm-specific TFs, which had been identified in our previous studies (Sandmann et al., 2006b, 2007; Jakobsen et al., 2007; Liu et al., 2009; Zinzen et al., 2009; Junion et al., 2012). One candidate TF was Ttk69, a repressor studied mainly in the context of early embryonic patterning (Brown and Wu, 1993) and nervous system development (Guo et al., 1995; Giesen et al., 1997; Badenhorst et al., 2002). We previously identified a region located ∼10 kb upstream of the *ttk* locus that is occupied by multiple mesoderm-specific regulators (Zinzen et al., 2009) (Fig. 1A). We also showed that this region is sufficient to drive expression in the developing visceral mesoderm (VM), which surrounds the gut (Jakobsen et al., 2007). Here, we demonstrate that the *ttk*-VME enhancer recapitulates the expression of the endogenous Ttk69 protein in somatic mesoderm (SM) and VM as well as in tracheal placodes at stage 11 (Fig. 1B,C) [staging according to Campos-Ortega and Hartenstein (1985)].

As a first step towards understanding the role of Tramtrack in muscle development, we analyzed its mesodermal expression in detail. Transcripts from the endogenous *ttk* locus are first detected at early stage 11 in the VM primordium (supplementary material Fig. S1A), and slightly later in a subset of cells within the SM (Fig. 1D). Whereas *ttk* expression is maintained in the VM until the end of embryogenesis (supplementary material Fig. S1B), its expression in the SM is very transient and is largely absent by stage 12 (Fig. 1E). This expression pattern is identical for both *ttk69* and *ttk88* isoforms (supplementary material Fig. S1D,E), consistent with observations in other tissues (Read and Manley, 1992). With a slight delay, the Ttk69 protein recapitulates the mRNA distribution in the VM and the underlying gut endoderm during stages 11 and 16 (Fig. 1F,G).

As the expression of Ttk69 in the SM is very transient, we took advantage of the stability of *green fluorescent protein* (*GFP*) in *ttk*-VME enhancer transgenic embryos to determine in which SM cell types *ttk* is expressed. Although the *ttk* enhancer drives expression in most, if not all, FCMs labeled by *lmd*, the majority of the FCs, marked by *dumbfounded* (*duf*; *Kirre* – FlyBase) are devoid of *GFP* transcripts (supplementary material Fig. S1C), indicating that *ttk* expression within the somatic muscle is largely FCM specific.

*Ttk69* therefore shows dynamic expression in the somatic FCMs during the stages of myoblast cell fate specification and robust and stable expression in the visceral mesoderm. This dynamic distribution indicates that *ttk* expression is subjected to tight transcriptional control and suggests that its misregulation could be detrimental to muscle development.

### The presence and timing of *ttk69* expression are essential for somatic muscle development

The *ttk69* amorphic allele *ttk^D2-50^*, previously classified among the strongest loss-of-function alleles (Giesen et al., 1997), most likely also affects the expression of the other *ttk* isoform, *ttk88*. However, a Ttk88-specific mutant (*ttk^1^*) is homozygous viable (Xiong and Montell, 1993) and embryos develop with no aberrant muscle development (data not shown). In all subsequent analyses, we therefore reasoned that any muscle phenotypes observed in *ttk^D2-50^* are primarily due to loss of the Ttk69 isoform.

**Fig. 2.**
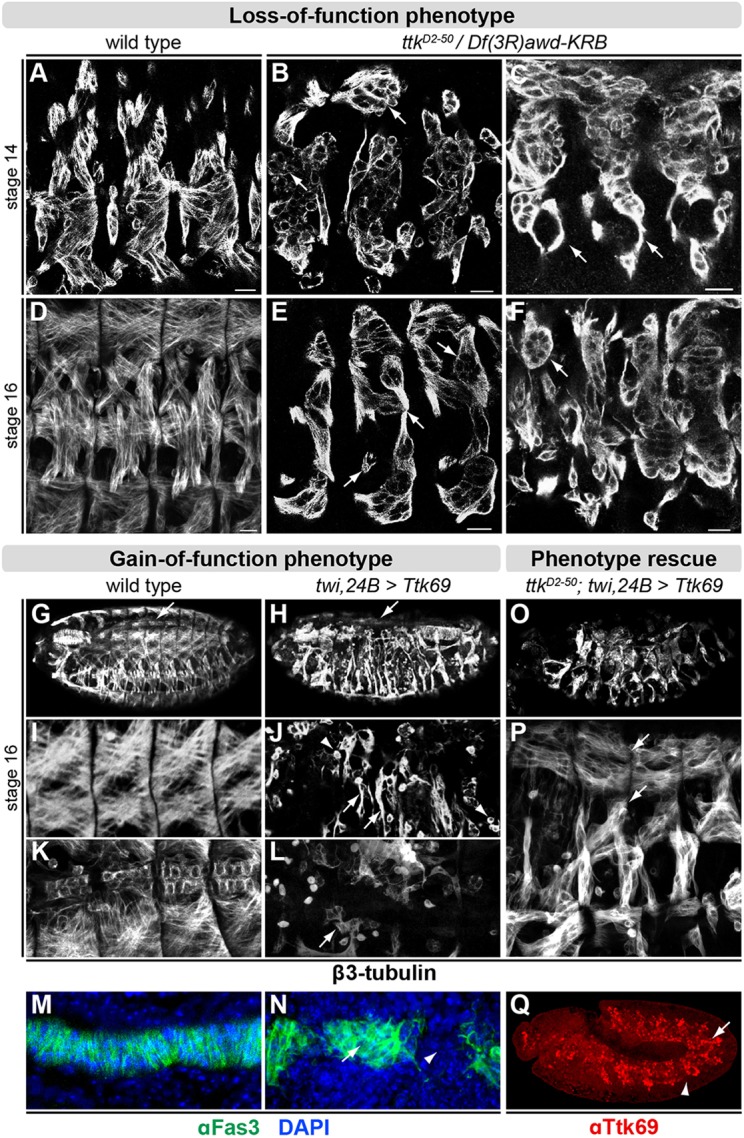
**Loss or gain of Ttk69 expression results in abnormal muscle development.** (A-F) Somatic musculature in wild-type and *ttk* mutant embryos [*ttk^D2-50^* in *trans* to *Df(3R)awd-KRB*] using an anti-β3-tubulin antibody. (A) At stage 14, wild-type mesodermal cells undergo myoblast fusion, leading to multinucleated muscle fibers. (B,C) Two different stage 14 Ttk69-deficient embryos in which myoblasts fail to fuse and form irregularly shaped aggregates (arrows). (D) Stereotypic organization of muscle fibers in three segments of a stage 16 wild-type embryo. (E,F) In stage 16 *ttk* mutant embryos, muscle fibers have abnormal morphology (arrows) and fail to form epidermal attachment sites. Scale bars: 10 µm. (G-L) Wild-type and ectopic Ttk69 embryos immunostained with anti-β3-tubulin antibody. (G) Heart (arrow) and somatic musculature in a stage 16 wild-type embryo. (H) In ectopic Ttk69 embryos, only a few isolated cardioblasts are formed (arrow) and the somatic musculature is disorganized. (I) High magnification of dorsal somatic musculature in a wild-type embryo. (J) Pan-mesodermal Ttk69 expression leads to thin muscle fibers without clear identity (arrows) and many mononucleated myoblasts (arrowheads). (K,L) High magnification of heart at stage 16 in wild-type (K) and ectopic mesodermal Ttk69 embryo (L), where only few cardioblasts are visible (arrow). (M,N) Visceral mesoderm stained with anti-Fas3 antibody (green) and DAPI (blue) at stage 13 in wild-type (M) and ectopic Ttk69 embryo (N), where cells fail to adopt columnar shape (arrow) and have visible gaps (arrowhead). (O-Q) Embryos with ectopic Ttk69 driven by *twi-Gal4, 24B-Gal4* in *ttk^D2-50^* mutant background. (O) Somatic musculature is disorganized, but to a lesser extent than with ectopic Ttk69 alone (compare with H). (P) High magnification of four segments shows fully extended dorsal and lateral muscle fibers (arrows) (compare with E,F,J). (Q) Immunostaining with anti-Ttk69 antibody at stage 11 with ectopic Ttk69 driven by *twi-Gal4, 24B-Gal4* in *ttk^D2-50^* mutant background. Ttk69 is only expressed in the mesoderm (arrow), whereas other sites of endogenous expression (e.g. tracheal placodes, arrowhead) lack Ttk69. All embryos are oriented anterior to the left and dorsal top.

Removing Ttk69 function by placing *ttk^D2-50^* in *trans* to a deficiency deleting the entire *ttk* locus [*Df(3R)awd-KRB*] caused severe somatic muscle defects. In wild-type stage 14 embryos, myoblast fusion is underway and the first muscle fibers have already formed (Fig. 2A). In *ttk* loss-of-function embryos, however, many muscle precursors remain mononucleated, with some aggregating into clusters (Fig. 2B,C), and fail to form the normal stereotypically organized muscle pattern (Fig. 2D-F).

In addition to the SM, the gut also displays abnormal morphology in *ttk* mutants, failing to form the characteristic three midgut constrictions (data not shown). Immunostaining for the visceral muscle-specific marker Fasciclin 3 (Fas3) (Patel et al., 1987) revealed that the number and morphology of visceral myoblasts are comparable to those of wild-type embryos (data not shown), suggesting that the gut phenotype is primarily due to a defect in endoderm rather than in visceral muscle development. Specification of heart precursors occurs normally (data not shown), although a normal dorsal vessel is never formed in *ttk^D2-50^* embryos, probably as a secondary consequence of a dorsal closure defect.

Two previous studies observed that *ttk* mutant embryos inappropriately express the neuronal protein Futsch (Hummel et al., 2000), the antigen of the 22C10 antibody (Zipursky et al., 1984), in somatic and visceral muscles (Giesen et al., 1997; Murawsky et al., 2001). We confirmed this observation and, in addition, found that Futsch is also misexpressed in the cardiac mesoderm (supplementary material Fig. S2), suggesting that *ttk* might be expressed at subdetectable levels in the heart and/or that Ttk69 induces non-cell-autonomous effects.

Given the very transient expression of *ttk69* in the SM, we investigated the effects of prematurely expressing Ttk69 throughout the mesoderm. For this purpose, we used the pan-mesodermal driver *twi-Gal4, 24B-Gal4* (Brand and Perrimon, 1993; Michelson, 1994) to induce expression of Ttk69 protein in the mesoderm by stage 9, prior to cell fate specification and approximately 3 h earlier than it is normally expressed. Whereas driving *UAS-GFP* or *UAS-ttk88* resulted in healthy adults, ectopic *ttk69* expression was lethal at embryonic stages as a result of severe defects in the specification of all three muscle types. The heart was either completely absent or only a few isolated cardioblasts were visible (Fig. 2K,L), while the visceral musculature contained gaps and had an abnormal organization (Fig. 2M,N). Within the somatic musculature, *ttk69* overexpression led to very few correctly specified and differentiated muscle fibers. Instead, the cells remained mononucleated and formed ‘mini-muscles’ that lack clear identity (compare Fig. 2I with 2J). The SM defects seem to arise from a cell-autonomous role of Ttk69 within the mesoderm, as expression of *ttk69* only in the mesoderm in a *ttk^D2-50^* loss-of-function mutant background (Fig. 2Q) can partially rescue defects in muscle morphology (Fig. 2O,P). The incomplete rescue is likely to be due to the gross differences in the spatiotemporal expression of the Gal4 driver compared with the endogenous *ttk69* gene. The observed ‘mini-muscles’ phenotype, induced from the ectopic expression of this transcriptional repressor, is very similar to the phenotype of *lmd* (Duan et al., 2001; Furlong et al., 2001; Ruiz-Gomez et al., 2002) and *Mef2* (Bour et al., 1995) loss-of-function mutants, two factors that act primarily as transcriptional activators.

These severe muscle phenotypes might be partially attributed to a role of Ttk69 in the regulation of *twist* expression, high levels of which are essential for normal SM development (Baylies and Bate, 1996). With ectopic Ttk69 expression we observed a severe reduction in the levels of *twist* transcripts (supplementary material Fig. S3A-F), and, conversely, in *ttk* loss-of-function embryos the number of Twist-expressing nuclei was elevated (supplementary material Fig. S3G-I). Given that Ttk69 binds to two regions in the *twist* locus (supplementary material Fig. S3J), these results suggest that Ttk69 might directly repress Twist expression during these stages of muscle development.

Taken together, these results indicate that the timing and transient nature of Ttk69 expression in FCMs are crucial for normal somatic muscle development. Given the rather unusual myoblast fusion phenotype in *ttk* mutants and its enriched expression in FCMs, we next examined whether Ttk69 is actively required during myoblast fusion, or whether the muscle phenotypes are due to its earlier role in myoblast cell fate specification.

### *ttk* mutants show a dramatic expansion of FC-like cells at the expense of FCMs

**Fig. 3.**
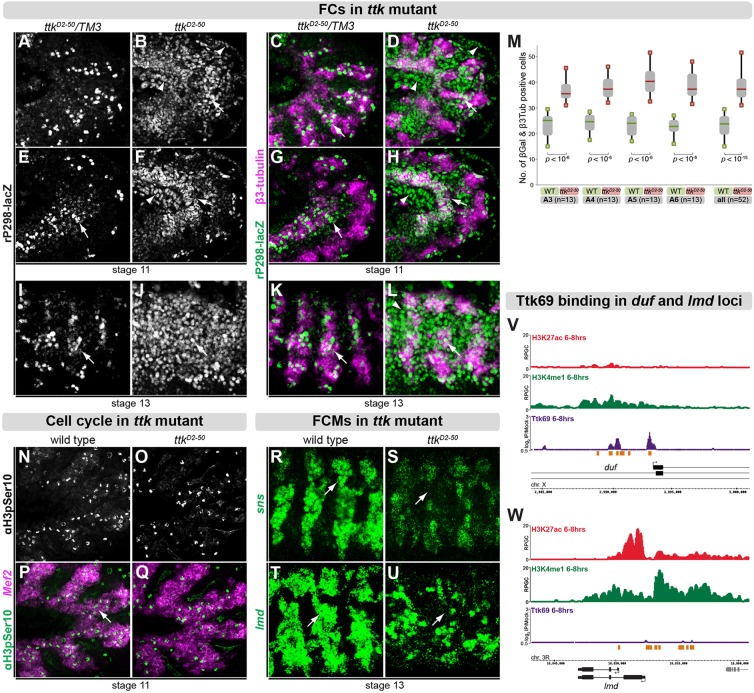
**Ttk69 is required for the balance between FCs and FCMs.** (A-L) Immunostaining against β-Gal driven by *rP298-lacZ* enhancer trap (gray, green) and the mesodermal marker β3-tubulin (magenta). At stage 11 (A-H) and 13 (I,L), β-Gal is strongly derepressed in *ttk^D2-50^* mutant embryos (B,F,J) compared with wild type (WT) (A,E,I). High magnification of three segments (stage 13) shows a drastic increase in cells marked by β-Gal in *ttk* mutants (J) compared with WT (I). Arrows point to mesodermal cells and arrowheads to non-mesodermal cells (D,H,L). (M) In *ttk^D2-50^* heterozygous (WT) and homozygous (*ttk^D2-50^*) embryos, cells expressing β-Gal and β3-tubulin in somatic mesoderm were quantified in four segments (A3-A6) at multiple focal planes. Per genotype, 52 segments in 13 different embryos were analyzed. *P-*values for significance were calculated using exact Wilcoxon rank sum test. (N-Q) Immunostaining against phosphorylated histone H3, marking mitotic cells (gray, green) combined with *in situ* hybridization against *Mef2*, marking mesodermal cells (magenta). At stage 11, no differences between WT (N,P) and *ttk^D2-50^* homozygous mutant (O,Q) embryos were detected. (R-U) Fluorescent *in situ* hybridization against *sns* or *lmd* (green) in WT (R,T) and *ttk* mutant (S,U) embryos. Expression of both genes is reduced in the absence of *ttk69* (arrows). High magnification images of three to four segments per embryo are shown. (V,W) Normalized log_2_ ChIP-chip signal (IP/mock) of Ttk69 binding (purple) and ChIP-Seq of mesoderm-specific profiles of chromatin marks H3K27 acetylation (red) and H3K4 monomethylation (green) (Bonn et al., 2012) in the *duf* (V) and *lmd* (W) loci. Mesodermal CRMs from Zinzen et al. (2009) indicated in orange. RPGC, reads per genomic coverage.

Each muscle fiber in *Drosophila* embryos is generated from a single FC that fuses to multiple FCMs, giving rise to a multinucleated myotube [reviewed by Rochlin et al. (2010)]. Duf, a transmembrane Ig-domain protein, together with its paralog *roughest* (Strunkelnberg et al., 2001), is expressed exclusively in FCs within the somatic mesoderm (Ruiz-Gómez et al., 2000); the *duf* enhancer trap line *rP298-lacZ* is therefore commonly used to track all FCs (Nose et al., 1998). To investigate whether the specification of FCs is affected in *ttk* mutant embryos, we placed *rP298-lacZ* into the *ttk^D2-50^* mutant background. In these *ttk* mutant embryos, the number of β-Galactosidase (β-Gal)-positive cells during stage 11 (before the onset of fusion), and during stage 13 (after the onset of fusion), is substantially higher compared with heterozygous embryos (Fig. 3A-L). Many cells outside of the mesoderm also express β-Gal (Fig. 3D,H,L), although this non-mesodermal expansion does not occur for the endogenous *duf* gene (data not shown).

To quantitatively assess the effect of loss of *ttk69* function on FC numbers, we manually counted cells positive for both β-Gal and the muscle-specific marker β3-tubulin in 13 different *ttk^D2-50^* heterozygous and homozygous embryos at stage 12 (Fig. 3M). Whereas the number of cells between four segments (A3-A6) within a single embryo did not vary substantially, *ttk* mutant embryos had significantly more β-Gal–β3-tubulin-positive myoblasts than wild-type embryos (mean 38.4 compared with 23.3; *P*<10^−15^, exact Wilcoxon rank sum test). The exact developmental identity of these ectopic mesodermal *rP298-lacZ-*positive cells is ambiguous, as the expression of FC identity proteins, such as Krüppel (Kr) (supplementary material Fig. S4A-D) or Even-skipped (data not shown), appears largely unaffected at stage 12. We therefore refer to these *rP298-lacZ*–*β3-tubulin*-expressing cells as ‘FC-like’, as they do not seem to activate a full specification program to convert them into FCs, but yet they have lost their potential to differentiate into FCMs. Upon myoblast fusion in wild-type embryos, FCMs have the capacity to acquire the identity and therefore the expression of FC-specific genes. The expanded expression of Kr and other FC identity genes at stage 15, when myoblast fusion is almost complete, is thereby a general indicator that FCMs are now part of a multinucleate syncytium. In *ttk* mutant embryos this expansion in Kr-positive cells does not occur (supplementary material Fig. S4E-I), indicating that these FC-like cells have a reduced capacity to undergo myoblast fusion, presumably due to their mixed identity.

Ttk69 is known to regulate both cell proliferation and cell cycle in multiple developmental contexts (Badenhorst, 2001; Jordan et al., 2006). However, the increased number of *rP298-lacZ*-positive myoblasts in *ttk* mutant embryos is not due to an aberrant cell cycle, as seen by immunostaining with a Histone H3 phospho-serine 10-specific antibody (Fig. 3N-Q), a commonly used mitosis marker (Hendzel et al., 1997).

Given that FCs and FCMs come from a common progenitor pool, we reasoned that the ectopic FC-like cells in *ttk* mutant embryos might be produced at the expense of FCMs. To examine the number of FCMs, we used *in situ* hybridization against two FCM-specific genes: *lmd* (Duan et al., 2001; Furlong et al., 2001; Ruiz-Gomez et al., 2002) and its direct target gene, *sns* (Ruiz-Gomez et al., 2002; Cunha et al., 2010). Both genes are expressed in fewer mesodermal cells in *ttk* mutant embryos than in wild-type embryos during stages 11-13 (Fig. 3R-U).

Taken together, these results indicate that, in the absence of Ttk69, FC-like cells are specified at the expense of FCMs. Given that Ttk69 is a well-established transcriptional repressor, this suggests that it is involved in the specification of FCM cell fate by counteracting or repressing an FC-specific developmental program within FCMs.

### Ttk69 and Lmd occupy largely distinct regions throughout the genome

To understand the molecular mechanism by which Ttk69 regulates FCM cell fate, we used chromatin immunoprecipitation (ChIP) to identify potential direct target genes of Ttk69. Two independent antibodies specific for the Ttk69 isoform were used to isolate Ttk69-occupied regions from embryos at 6-8 h after fertilization (stages 10 and 11), a time window spanning mesodermal subdivision and SM specification. The bound fragments were hybridized to high-density whole-genome *Drosophila* tiling arrays and analyzed as previously described (Zinzen et al., 2009).

**Fig. 4.**
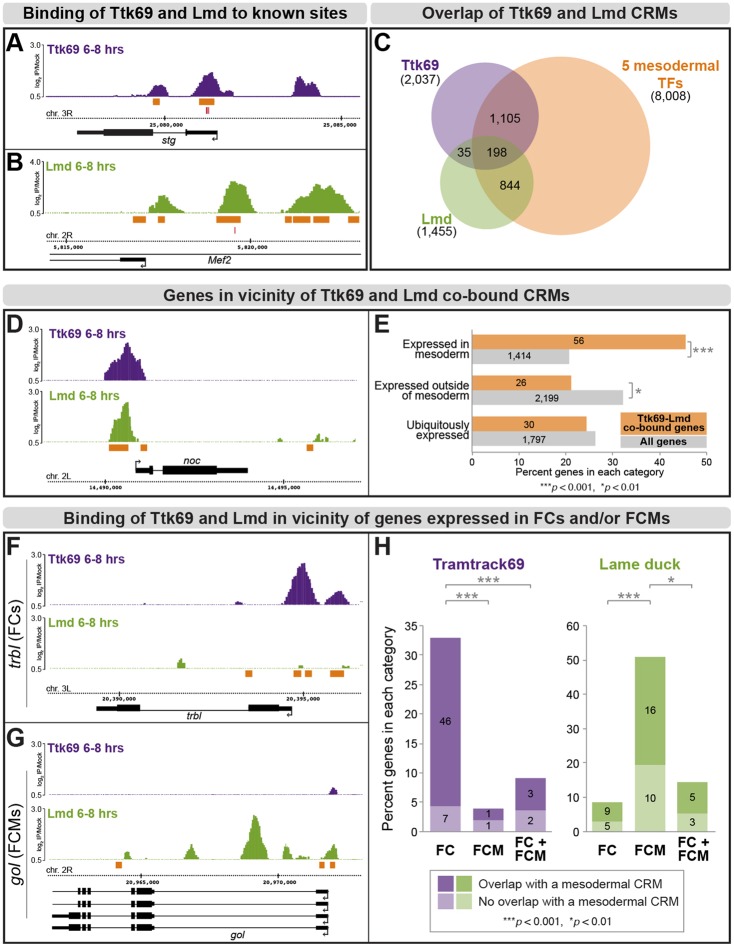
**Genome-wide Ttk69 and Lmd binding activity.** (A,B) Binding profiles [normalized log_2_ ChIP signal (IP/mock)] of Ttk69 (A, purple) and Lmd (B, green) in loci of their known target genes *stg* and *Mef2*, respectively. Mesodermal CRMs in orange and experimentally validated binding sites in red. (C) Venn diagram showing overlap of regions bound by Ttk69 (purple), Lmd (green) or five key mesodermal TFs (orange). (D) Locus of a representative mesodermal gene, *noc*, with Ttk69 (purple) and Lmd (green) ChIP signal (log_2_ IP/mock) and mesodermal CRMs (orange) upstream of its TSS. (E) BDGP database survey of expression of genes associated with 233 Ttk69-Lmd co-bound CRMs. Orange indicates genes with at least one Ttk69-Lmd co-bound CRM in their proximity and gray designates all genes annotated by BDGP. The gene numbers are indicated. (F,G) Ttk69 (purple) binds to the FC-specific gene *trbl* (F) and Lmd (green) occupies multiple CRMs in the locus of *gol*, an FCM-specific gene (G). (H) Global analysis of Ttk69 and Lmd binding preferences within 1.5 kb of TSS of differentially expressed genes. Dark purple and green represent genes with at least one CRM overlapping a mesodermal CRM; light colors indicate genes where CRMs do not overlap mesodermal CRMs. *P-*values calculated using Fisher's exact test.

We identified 2037 high-confidence Ttk69-bound regions [putative *cis*-regulatory modules (CRMs)] (supplementary material Table S1). Although there are no known direct Ttk69 targets at these stages, the expression of ten genes responds to Ttk69 in the embryonic nervous system or trachea at stages 10 and 11. Eight of these genes have high-confidence Ttk69-bound regions in their vicinity (Fig. 4A; supplementary material Fig. S5), with a ninth gene, *mmy*, being just below our stringent cut-off, suggesting that these genes are directly regulated by Ttk69. An additional 13 genes are repressed by Ttk69 at developmental stages outside of the time window assayed. Significant Ttk69 binding was observed at all 13 loci (supplementary material Fig. S5), suggesting that Ttk69 might act as a constitutive repressor of the expression of these genes. *De novo* motif analysis within 100 bp of Ttk69-bound peaks revealed a significant enrichment in the Ttk69 motif, as expected, and, interestingly, also in a bHLH motif that matches the preferred motif of Nautilus (supplementary material Fig. S6), a muscle-specific TF (Balagopalan et al., 2001; Wei et al., 2007). This finding, taken together with the high percentage (91%: 21/23) of recovered genes known to be genetically regulated by Ttk69, underscores the sensitivity of the data and provides a global view of Ttk69 regulatory input.

Ttk69 binds to multiple regions in the introns and upstream of *duf* (Fig. 3V) at the approximate site of the *rP298* P-element insertion (Ruiz-Gómez et al., 2000). By contrast, there was no detectable binding in the *lmd* locus (Fig. 3W), suggesting that this essential FCM-specific gene is not directly regulated by Ttk69. Both results reinforce our hypothesis that the decreased number of FCMs in *ttk* mutants is due to their partial conversion to FC-like fates through the derepression of a subset of FC genes.

As this model points to a role of Ttk69 in FCM specification, we examined the genome-wide occupancy of the FCM-specific TF Lmd. Genome-wide ChIP analysis performed under identical conditions identified 1455 high-confidence Lmd-bound regions (supplementary material Table S2). These include the known Lmd-regulated enhancer within the *Mef2* locus (Duan et al., 2001) (Fig. 4B), as well as high-confidence peaks within seven of ten previously characterized regions that respond to Lmd *in vivo* and/or *in vitro*, with an eighth gene, *CG5080*, being just below our stringent cut-off (Cunha et al., 2010) (supplementary material Fig. S7).

Bound regions identified in both Ttk69 and Lmd experiments significantly overlap with the 8008 CRMs identified in our previous ChIP studies bound by five mesoderm-specific TFs (Zinzen et al., 2009) (referred to as mesodermal CRMs): 64% (1303/2037) for Ttk69 and 72% (1042/1455) for Lmd (Fig. 4C; supplementary material Table S3). Over 90% of these regions have mesodermal activity when tested *in vivo* (Zinzen et al., 2009), which implies that a substantial portion of Ttk69 and Lmd binding occurs within mesodermal enhancers. Comparing the occupancy of each TF with each other revealed that the majority of Ttk69-bound regions (89%: 1804/2037) and Lmd-bound regions (83%: 1222/1455) do not overlap (Fig. 4C; supplementary material Table S3), indicating that the bulk of their regulatory input occurs through different *cis*-regulatory modules, targeting largely different sets of genes as discussed below.

Interestingly, there are 233 CRMs that are bound by both factors (Fig. 4C) and, although the overlap is small, it is highly significant compared with randomly reshuffled genomic regions (*P*<10^−320^; Fig. 4C). To examine the expression of the associated genes, we assigned each CRM to the nearest transcriptional start site (TSS) and surveyed the Berkeley Drosophila Genome Project (BDGP) *in situ* hybridization database (Tomancak et al., 2007) (Fig. 4D,E; supplementary material Table S4). Of the 214 unique genes, the expression of 123 genes is annotated, of which 30 genes (24%) are expressed ubiquitously. Of the 93 genes with tissue-specific embryonic expression, 56 (46% of 123 genes) are expressed in mesoderm or its derivatives during at least one stage of embryonic development. This represents a significant enrichment (*P*<5.6×10^−10^, two-sided Fisher's exact test) over the expected number of mesodermal genes in the entire set of BDGP annotated genes (1414/6835 genes), whereas the number of genes expressed outside of the mesoderm is significantly depleted (21% compared with 32%, *P*<0.009, two-sided Fisher's exact test). These data indicate that these two TFs are not co-binding to random regions, but rather on a selected number of regulatory elements in the vicinity of a subset of mesodermal or muscle genes, and suggest that Ttk69 might have an additional role in controlling mesodermal gene expression within or outside of the mesoderm.

### Ttk69 binds to enhancers of FC-specific genes, whereas Lmd targets FCM-specific genes

As described above, the vast majority (over 80%) of regions bound by Ttk69 or Lmd do not overlap, despite the binding profiles of each factor having almost 50% overlap with mesodermal CRMs (Fig. 4C). As Ttk69 is a well-established repressor and Lmd acts predominantly as an activator (Cunha et al., 2010), we reasoned that they regulate distinct sets of target genes within FCMs. We therefore examined genes in the vicinity of Ttk69-only or Lmd-only CRMs for their expression in either FCs or FCMs. We used data from *in situ* hybridization experiments that classified the expression of about 300 genes as FC specific, FCM specific or both (Estrada et al., 2006). We then searched within ±1500 bp of the TSS of each gene for Ttk69-only or Lmd-only CRMs (supplementary material Table S5). For example, a region overlapping two mesodermal CRMs upstream of *tribbles* (*trbl*), an FC-specific gene, is highly bound by Ttk69 (Fig. 4F), whereas there is no significant Lmd binding. Conversely, there is no significant Ttk69 binding near the FCM-specific gene *goliath* (*gol*) (Cunha et al., 2010), yet Lmd binds upstream of the TSS as well as in an intronic region (Fig. 4G). Globally, almost 33% (53 out of 161) of known FC-specific genes contain Ttk69 bound CRMs with no Lmd-bound regions in their vicinity, compared with only ∼4% of FCM-specific genes or ∼9% of genes expressed in both FCs and FCMs (Fig. 4H). The vast majority of these Ttk69 binding events (87%: 46 out of 53) are within regions occupied by mesodermal TFs, suggesting that these are active mesodermal enhancers. By contrast, Lmd has a highly significant binding preference for loci of FCM-specific genes; 51% of known FCM-specific genes have an Lmd bound CRM and no Ttk69 binding in their vicinity compared with only 9% of FC-specific genes (Fig. 4H; supplementary material Table S5).

We next asked if Ttk69-bound and Lmd-bound CRMs have differential activity, using chromatin state as a readout of enhancer activity. We used mesoderm-specific ChIP-Seq profiles of H3K4me1 [marking both active and inactive CRMs (Bonn et al., 2012)], H3K27ac [predictive of active CRMs (Bonn et al., 2012)] and H3K27me3 [indicating a Polycomb repressed state (Bonn et al., 2012)] at 6-8 h of embryogenesis, the same time window as the Ttk69 and Lmd ChIP experiments. We note that although the chromatin data are mesoderm specific, FCMs represent a relatively small population of cells within the mesoderm. We focused on CRMs and TSSs in the vicinity of FC and FCM genes for Ttk69 and Lmd, respectively. H3K4me1 has a similar distribution at Ttk69 and Lmd CRMs, as expected for a chromatin mark constitutively associated with regulatory elements (supplementary material Fig. S8A). By contrast, H3K27ac is differentially enriched at Lmd CRMs and their associated TSSs, compared with Ttk69; this is consistent with their role in transcriptional activation or repression, respectively, as observed globally (supplementary material Fig. S8A,B) and shown for the *lmd* (Fig. 3W) and *duf* (Fig. 3V) loci. The repressive mark H3K27me3 is largely absent from both groups of elements, indicating that the Polycomb system does not play a role in Ttk69-mediated repression.

Thus, Ttk69 and Lmd display distinct binding profiles at CRMs with different chromatin states in the vicinity of genes differentially expressed in the two populations of fusing myoblasts. Ttk69 is mainly bound to inactive enhancers associated with genes exclusively expressed in FCs. As Ttk69 is a transcriptional repressor, and as the expression of an FC-specific reporter *rP298-lacZ* is derepressed in FCMs in *ttk* mutants (Fig. 3), these results suggest that Ttk69 contributes to FCM cell fate by directly repressing a substantial part of an FC-transcriptional program in FCMs. Conversely, Lmd binding is preferentially associated with enhancers in an active state, which are located in the vicinity of genes expressed in FCMs.

### Ttk69 represses enhancer activity during mesoderm development

**Fig. 5.**
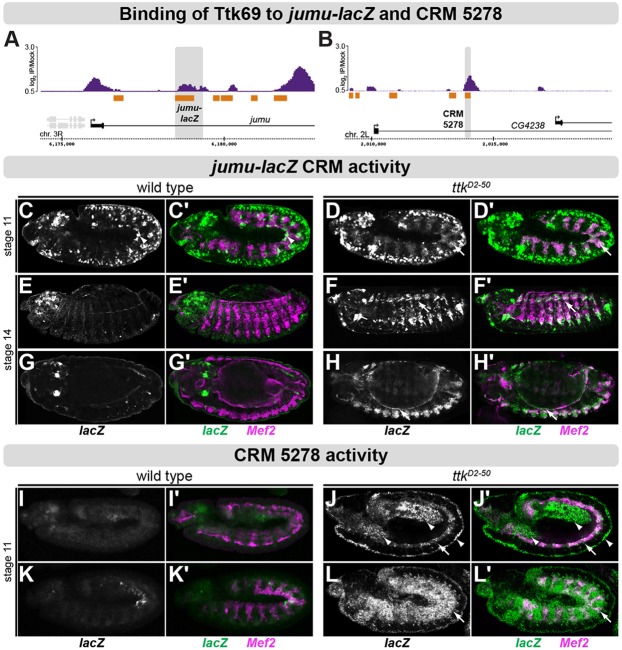
**Ttk69 is essential for restricting CRM activity across cell and tissue types.** (A,B) Ttk69 binding [normalized log_2_ ChIP signal (IP/mock)] within the *jumu* (A) and *CG4238* (B) loci. Mesodermal CRMs indicated in orange and cloned regions in gray. (C-H) Double fluorescent *in situ* hybridization against *jumu-lacZ* (gray, green) and *Mef2* (magenta) in wild-type and *ttk^D2-50^* mutant embryos. At stages 11 and 14 in wild-type embryos (C,C′,E,E′,G,G′), no mesodermal activity is detected (with the exception of caudal VM, arrowhead). (D,F,H) In *ttk^D2-50^* homozygous mutant embryos, expression of *jumu-lacZ* is present in somatic mesoderm (arrows) at stage 11 (D,D′) and 14 (F,F′) and in VM (arrows) at stage 14 (H,H′). (I-L) *In vivo* activity of CRM *5278-lacZ* (gray, green) and *Mef2* (magenta) in wild-type and *ttk^D2-50^* mutant embryos. (I,I′,K,K′) In wild-type embryos, CRM *5278-lacZ* is active only in the caudal VM and head region at stage 11. (J,J′,L,L′) In *ttk69*-deficient embryos, *5278-lacZ* is active in multiple tissues, including visceral and somatic mesoderm (arrows), ectoderm and endoderm (arrowheads). Embryos are oriented with anterior to the left and dorsal top. All embryos are shown in lateral views, with the exception of G and H, which are dorsolateral.

To confirm that Ttk69 is essential to repress enhancers within FCMs, we examined the activity of two Ttk69-bound regions within the *jumeau* (*jumu*) and *CG4238* loci (Fig. 5). *jumu* has a dynamic expression in the ectoderm and nervous system, where it is essential for neuroblast identity (Cheah et al., 2000), and in the somatic mesoderm, where it has been reported to have FC-specific expression (Estrada et al., 2006). *In situ* hybridization shows weak *jumu* expression in the somatic mesoderm in *duf-*positive cells prior to myoblast fusion (supplementary material Fig. S9A), although it is difficult to say whether this expression is FC-specific given its rather weak and transient nature. The Ttk69-bound region was linked to a *lacZ* reporter gene, stably integrated into the *Drosophila* genome, and its activity was assayed in transgenic embryos by monitoring reporter gene expression. In wild-type embryos, the *jumu-lacZ* CRM is transiently active in the mesoderm at stage 6 (supplementary material Fig. S9), becomes highly expressed in the neuroectoderm, caudal visceral mesoderm (cVM) and tracheal placodes at stage 11 (Fig. 5C,C′), and continues to be active in discrete neuroectodermal cells and central nervous system (CNS) at later stages (Fig. 5E-G). With the exception of cVM, this enhancer is not active in the mesoderm and its derivatives after stage 6 (Fig. 5; supplementary material Fig. S9). In *ttk* mutants, however, the *jumu-lacZ* CRM showed a striking derepression in the somatic mesoderm, starting at stage 11 (compare Fig. 5C,C′ with 5D,D′) and increased at stage 14 (compare Fig. 5E,E′ with 5F,F′). The CRM is also derepressed in the visceral muscle (compare Fig. 5G,G′ with 5H,H′), and in other tissues where Ttk69 is expressed (supplementary material Fig. S9).

A similar mesodermal derepression was observed for CRM *5278-lacZ* (Zinzen et al., 2009), located in the intron of *CG4238*. This enhancer is bound by Ttk69 at stages 10 and 11 (Fig. 5B) and has transient, early activity in the mesoderm up until stage 10 (Zinzen et al., 2009). After stage 11, *5278-lacZ* is inactive throughout the embryo, with the exception of cVM and two patches of activity in the head region (Fig. 5I,I′,K,K′) (Zinzen et al., 2009). In *ttk* mutant embryos, however, the enhancer shows a dramatic derepression (Fig. 5J,J′,L,L′), in both the somatic and visceral mesoderm, as well as in the ectoderm and endoderm (compare Fig. 5I,I′ with 5J,J′ and 5K,K′ with 5L,L′). Similarly, the *CG4238* gene is derepressed in multiple tissues, such as endoderm, salivary gland and a subset of somatic mesoderm from stage 11 (supplementary material Fig. S10).

These results, combined with the *in vivo* binding data, indicate that Ttk69 acts as a potent repressor during mesoderm development, being required to restrict enhancer activity in cells of both the somatic and visceral mesoderm, as well as in other embryonic tissues.

## DISCUSSION

By combining genetic and genomic approaches, we have uncovered a novel role for a well-studied transcriptional repressor, Ttk69, in establishing FCM cell fate. Despite its very transient expression in somatic mesoderm, Ttk69 is essential for myogenesis; in *ttk* mutant embryos a pool of FC-like cells is expanded at the expense of FCMs. Genome-wide TF occupancy analysis provides a molecular explanation for how this severe muscle phenotype arises: Ttk69 binds predominantly to CRMs in the vicinity of FC-specific, but not FCM-specific, genes, where it represses their activity. By contrast, the FCM TF Lmd predominantly occupies CRMs in the vicinity of FCM-specific genes. As observed in other developmental contexts, the specification of FCMs requires more than the simple deployment of a single gene regulatory network, but rather the simultaneous activation and repression of two distinct transcriptional programs.

### Could Ttk69 act downstream of Notch signaling to regulate FCM cell fate?

**Fig. 6.**
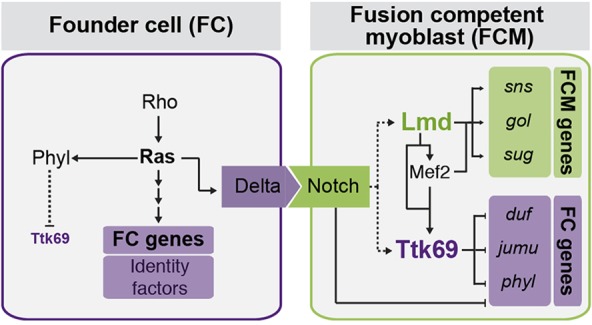
**A model of Ttk69 function in FCM specification.** Based on our data, Ttk69 acts as a repressor of FC fate, whereas Lmd activates the expression of FCM-specific genes, potentially including *ttk69* itself. Ttk69 is downstream of Notch signaling in multiple systems, suggesting that Notch might also positively regulate *ttk69* expression in FCMs. In FCs, Ras signaling initiates expression of FC-specific genes, such as *phyllopod* (*phyl*), a negative regulator of Ttk69 protein levels.

Similar to its function in the embryonic nervous system, Notch signaling promotes FCM fate by blocking the acquisition of FC fate in surrounding myoblasts (Bour et al., 2000). In the nervous system, Ttk69 has been shown to repress neuronal cell fate in glial cells and to genetically interact with Notch (Guo et al., 1996; Giesen et al., 1997). It is thus possible that Ttk69 is a mechanistic link between Notch and its responsive genes in FCMs: Notch signaling might activate Ttk69 expression in FCMs as discussed below. Ttk69, once expressed, then acts to repress an FC-specific transcriptional program, while allowing the FCM program to proceed (Fig. 6). The relationship between Notch signaling and Ttk69 activity might be more complex, as a negative-feedback loop from Ttk69 to Notch has been observed in follicle cells (Boyle and Berg, 2009) and, most likely, also in tracheal cells (Rotstein et al., 2011). In agreement with this, our ChIP data identified *in vivo* Ttk69 binding in the vicinity of multiple genes encoding components of the Notch pathway (data not shown), indicating that Ttk69 has at least the potential to contribute to negative-feedback regulation directly. Although speculative, the many links between Notch signaling and Ttk in other contexts, including the fact that ectopic Notch signal is sufficient to induce Ttk expression in the peripheral nervous system (Guo et al., 1996), suggest that Ttk might be responsive to Notch signaling in FCMs as well.

### Regulation of Ttk69 activity specifically in FCMs

How is Ttk69 activity regulated in FCMs? In addition to a potential induction of Ttk69 expression in FCMs by Notch signaling (discussed above), data presented here and in our previous study suggest that Ttk69 is directly activated by Lmd. A number of CRMs in the *ttk* locus are bound by Lmd at stages 10 and 11 (supplementary material Fig. S7B), and Lmd together with Mef2 cooperatively activate one of these enhancers in *Drosophila* S2 cells (Cunha et al., 2010). Ttk69 activity is most likely also restricted to FCMs by post-translational downregulation of any low-level *ttk69* expression in FCs: the Ttk69 protein contains stretches of PEST sequences, a hallmark of a short half-life (Rogers et al., 1986; Harrison and Travers, 1990), and its levels are dynamically regulated by proteasome-mediated degradation (Li et al., 1997; Cooper et al., 2008). This degradation requires Phyllopod (Li et al., 1997), an E3 ligase adaptor protein, the expression of which we previously showed to be enriched in FCs compared with FCMs (Artero et al., 2003). In the somatic mesoderm, Ttk69 activity is therefore most likely restricted to FCMs at both transcriptional and post-translational levels.

Taken together, our findings indicate that the concurrent activation and repression of an opposing cell fate is required to promote FCM cell identity during *Drosophila* myogenesis. We also identified Ttk69 as a novel myogenic TF playing a crucial role in this process.

## MATERIALS AND METHODS

### Fly stocks

The following *Drosophila* lines were used: *ttk^D2-50^* (C. Klämbt, University of Münster, Germany); *Df(3R)awd-KRB* (Bloomington Stock Center, USA); *twi-Gal4, 24B-Gal4* (M. Baylies, SKI, USA); *ttk^1^*, *UAS-Ttk69* and *UAS-Ttk88* (A. Travers, MRC, UK). *rP298-lacZ* (M. Ruiz-Gómez, CBM, Spain), *jumu-lacZ* (this study) and *5278-lacZ* (Zinzen et al., 2009). For the rescue experiments, *twi-Gal4, 24B-Gal4* and *UAS-Ttk69* were recombined with *ttk^D2-50^*.

### Generation of transgenic reporter lines

The DNA fragment covering the *jumu* CRM (dm3/BDGP release 5.0 chr3R: 6,178,511-6,179,367) was subcloned into a pH-Pelican vector for germline transformation (Barolo et al., 2000). Three independent fly lines were established and tested to exclude positional effects.

### *In situ* hybridization and immunostaining

Fluorescent *in situ* hybridization and antibody staining were performed as described previously (Furlong et al., 2001). The following ESTs were used to generate antisense probes: LD47926 (*lmd*), LD47926 (*twi*) and GM09101 (*ttk*). Full-length cDNA clones of *sns*, *Mef2* and *lacZ* were kind gifts from S. Abmayr (Stowers Institute for Medical Research, Kansas City, USA), M. Taylor (Cardiff University, Cardiff, UK) and M. Treier (Max-Delbrück-Center for Molecular Medicine, Berlin, Germany), respectively. The probes were detected with peroxidase-conjugated antibodies (Roche) and developed using the TSA system (PerkinElmer). *ttk* mutant embryos were unambiguously identified based on the absence of expression from the balancer chromosome.

The following primary antibodies were used at indicated dilutions: chicken anti-β-Galactosidase 1:300 (ab9361; Abcam), mouse anti-GFP 1:300 (ab1218; Abcam), rabbit anti-β3-tubulin 1:300 (Leiss et al., 1988), rabbit anti-Mef2 1:200 (Sandmann et al., 2006b), rabbit anti-Ttk69 1:200 (gift from F. Azorin, Institute for Research in Biomedicine, Barcelona, Spain), mouse anti-Bin 1:50 (J.S. Jakobsen, PhD thesis, University of Copenhagen, 2007), rabbit anti-phospho-histone H3 (Ser10) 1:200 (06-570; Millipore), mouse anti-Futsch 1:50 (22C10; DSHB), mouse anti-Fas3 1:5 (7G10; DSHB), guinea pig anti-Kr 1:100 and guinea pig anti-Eve 1:200 (gift from H. Jäckle, Max Planck Institute for Biophysical Chemistry, Göttingen, Germany).

### ChIP-on-chip and data analysis

ChIP was carried out according to Sandmann et al. (2006a). Two polyclonal Ttk69 antibodies [gifts from F. Azorin (Pagans et al., 2004) and A. Travers (Lehembre et al., 2000)] and one Lmd-antibody (Cunha et al., 2010) were used to immunoprecipitate Ttk69 or Lmd-bound fragments from 6- to 8-h-old wild-type embryos, respectively. Three independent ChIP and mock immunoprecipitations (IPs), using rabbit pre-immune serum, were performed. Purified DNA fragments were PCR amplified and hybridized to an Affymetrix GeneChip *Drosophila* tiling array 1.0R, as described (Sandmann et al., 2006a).

Bioinformatics analysis was performed as described (Zinzen et al., 2009). After data normalization, significantly enriched regions were determined using TileMap (Ji and Wong, 2005). For each significant region, the ChIP peak was calculated and CRMs defined as a 200 bp region centered around the peak (Zinzen et al., 2009). All ChIP raw data are available in ArrayExpress [accession numbers E-MTAB-1287 (Ttk69) and E-MTAB-1283 (Lmd)] and bed files for visualization are available at http://furlonglab.embl.de/data/.

### *De novo* motif discovery

*De novo* motif discovery in Ttk69 regions was performed with XXmotif (Luehr et al., 2012) using 200 bp repeat-masked regions centered on the ChIP peak. TOMTOM (Gupta et al., 2007) was used (default parameters, but AT/GC content was set to 0.3/0.2) to match discovered motifs to known *Drosophila* PWM databases (FlyFactorSurvey, FlyRegv2, idmmpmm2009 and dmmpmm2009) with *P*-value ≤0.05.

## Supplementary Material

Supplementary Material
