# Supplementary material for: Coordinated repression and activation of two transcriptional programs stabilizes cell fate during myogenesis
Source: Development. 2014 Jul;141(13):2633–43. doi: 10.1242/dev.101956 (PMC4146391; doi:10.1242/dev.101956)
Supplement: Supplementary Material [file supp_141_13_2633__index.html]

Coordinated repression and activation of two transcriptional programs stabilizes cell fate during myogenesis — Supplementary Material 

# Coordinated repression and activation of two transcriptional programs stabilizes cell fate during myogenesis

## DEV101956 Supplementary Material

**Files in this Data Supplement:**

- **Supplementary Material**
