# Supplementary material for: Coordinated repression and activation of two transcriptional programs stabilizes cell fate during myogenesis
Source: Development. 2014 Jul;141(13):2633–43. doi: 10.1242/dev.101956 (PMC4146391; doi:10.1242/dev.101956)
Supplement: Supplementary Material [file supp_141.13.2633_DEV101956.pdf]

Figure S1. Endogenous *ttk* transcripts are stably expressed in the developing visceral mesoderm

Double *in situ* hybridization against *ttk* (green) and *Mef2* (red). DAPI was used to stain nuclei (blue). (**A-A**'') Co-expression of *ttk* and *Mef2* at early stage 11 in the visceral mesoderm primordium (arrows). *ttk* is also expressed in tracheal placodes (arrowheads). (**B-B**'') *ttk* and *Mef2* continue to co-localize in the visceral muscle at stage 15 (arrows). (**C-C**''') Stage 11 *ttk*-VME transgenic embryo stained by *in situ* hybridization against *GFP* (green), *lmd* (red) and *duf* (blue). Arrows point to a *duf*-positive FC that is negative for *lmd* (marker of FCM) and *GFP*. (**D**) Schematic representation of the 8 different mRNA isoforms arising from the *ttk* locus. Regions that were used to generate protein isoform specific *in situ* probes against *ttk69* and *ttk88* are indicated in red and green, respectively. (**E-E**''') Triple fluorescent *in situ* hybridization against *ttk69*, *ttk88* and *Mef2* (mesodermal marker). In (**E**'''), merge between all three probes is shown, highlighting their co-localization in the visceral mesoderm (arrow) and somatic muscle primordia.

Figure S2. Neuronal marker 22C10 labels all muscle types in *ttk* mutants

Fluorescent immunostain with the antibodies 22C10 (anti-Futsch, green) and anti-Mef2 (magenta). Futsch and Mef2 are co-expressed in the somatic (**A**), visceral (**A**'), and cardiac (**A**'') muscle of stage 13 embryos. Note that Futsch is a cytoplasmic, microtubule-associated protein while Mef2 is located in the nucleus.

Figure S3. *twist* expression is regulated by Ttk69 activity in the mesoderm

(**A-F')** Expression of *twist* (green) was analyzed by fluorescent *in situ* hybridization in wild type embryos and in embryos with ectopic panmesodermal *ttk69* expression (red). During early stages before the onset of ectopic Ttk69 expression (**B**' and **D**'), *twist* is strongly expressed in both genotypes (**A-D**'). At stage 9, when ectopic *ttk69* transcripts accumulate in the mesoderm (**F**'), expression of *twist* is significantly reduced (**F**) compared to wild type embryos (**E**). (**G-I') Double immunostain of a *ttk* mutant embryo with a Twi-specific antibody (red) and an antibody directed against β3-tubulin (green). In wild typo embryos (**G** and **G**'), there are six adult muscle precursor cells expressing Twi in each hemisegment (arrows). In *ttk* loss-of-function mutant background, of which two representative embryos are shown (**H** and **H**', **I** and **I**') there are often more then 6 Twi-positive cells observed (arrows). Note that the relative position of the cells within the hemisegment is also affected. (**J**) Ttk69 (purple) binding signal (normalized $\log_2$ ChIP-on-chip signal (IP/mock)) in the *twist* locus. In orange are previously identified mesodermal CRMs.

Figure S4. Expression of the muscle identity gene Kr in *ttk* mutant embryos

Expression pattern of Kr was visualized by immunostain with anti-Kr antibody (shown in gray and red) in combination with an antibody against β3-tubulin (green), labeling all muscle cells. (**A-D**) At stage 12, before the onset of myoblast fusion, the number of Kr-positive cells is similar in wild type (**A** and **B**) and *ttk* mutant (**C** and **D**) embryos. (**E-H**) At stage 15, when myoblast fusion is almost finished, there is a decrease in the number of cells expressing Kr in *ttk* mutant embryos (**G** and **H**) compared to wild type embryos (**E** and **F**). (**I**) Quantification of the number of Kr-expressing nuclei: while in stage 15 wild type embryos (gray) there are 18 to 21 Kr-positive cells in each region (dorsal, lateral and ventral), in *ttk* mutant embryos (red) the number decreases to 9 to 12 cells. For each genotype, average value of Kr and β3-tubulin positive cells in 20 hemisegments in at least five different embryos is shown. The error bars indicate standard deviation.

Figure S5 (part 3). Ttk69 binding in the loci of its known targets

Normalized Ttk69 ChIP-on-chip signal in the vicinity of genes previously associated with Ttk69 activity at different stages of *Drosophila* development: *stg* (**A**), *dap* (**B**), *rap* (**C**), and

ci (**D**) in the oocyte; *ftz* (**E**), *eve* (**F**), *tll* (**G**), *run* (**H**), *h* (**I**), *odd* (**J**) in the syncytial blastoderm; *esg* (**K**), *mmy* (**L**), *pyd* (**M**), *bnl* (**N**), *scrt* (**O**), *repo* (**P**), *dpn* (**Q**), *futsch* (**R**) in the embryo; *ase* (**S**) and *CycE* (**T**) in the embryo and larva; and *ac* (**U**), *sc* (**V**), and *lz* (**W**) in the larva. Note that only two genes (*repo* and *mmy*) lack significant Ttk69 binding in their proximity, with *mmy* being just below our stringent cut-off. Previously identified mesodermal CRMs (Zinzen et al., 2009) are indicated in orange.

Figure S6. *De novo* motif discovery within Ttk69-bound ChIP peaks
Position Weight Matrices (PWMs) reported by XXmotif with an E-value ≤1e-3 and matching at least 5% of the input sequence are shown in the left column (individual XXmotif reported E-value is indicated below each sequence logo). The TOMTOM tool (Balagopalan et al., 2001) was used to systematically match discovered motifs to known *Drosophila melanogaster* PWM databases. Motif matches with q-value ≤ 0.05 (with the exception of Nau with q-value 0.06) are listed in the right column. Ttk was identified (first row) together with Nau, and additional factors with very repetitive motifs, such as Trl, Aef1 and Jim.

Figure S7 (part 2). Lmd binding in the loci of its known targets
Overview of Lmd binding in the loci of previously identified Lmd responsive genes (Cunha et al., 2010): *sns* (**A**), *ttk* (**B**), *blow* (**C**), *Act57B* (**D**), *®Tub60D* (**E**), *sug* (**F**), *CG14687* (**G**), *CG5080* (**H**), *Tret1-1* (*CG30035)* (**I**), and *CG9416* (**J**). Previously identified mesodermal CRMs (Zinzen et al., 2009) are indicated in orange. In gray (**B**) is the *ttk*-VME enhancer driving expression in visceral and somatic mesoderm (Jakobsen et al., 2007).

Figure S8. Differential chromatin state at Ttk69 and Lmd-bound regions
The distribution of the quantitative ChIP-Seq signal for three chromatin modifications is shown across bound regions in the vicinity of FC or FCM genes. Spatial distribution of mesoderm-specific signal for the H3 modifications K4me1, K27ac, and K27me3 at 6-8 hrs on enhancers (**A**) and their closest TSS (**B**). X-axes indicate distance from CRM centre (**A**) or from the TSS (**B**), y-axes show background subtracted signal. Shaded areas indicate the 95% confidence intervals of the signal. Note that H3K27ac, a chromatin modification associated with activation, is highly enriched at both Lmd bound CRMs and associated TSS. H3K27me3, a chromatin modification associated with Polycomb mediated repression, is not enriched in any class, indicating that Polycomb is not associated with Ttk mediated repression.

Figure S9. Activity of the *jumu* enhancer in wild type and *ttk* mutant embryos
(A-B') Expression of the gene *jumu* (gray, green) partially overlaps *duf* (magenta), a marker of all founder cells. **B** and **B**' are high magnification images of the embryo in **A** and **A**'. The embryo is orientated in lateral view. Arrows point to founder cells. **(C-H')** Fluorescent *in situ* hybridization against *lacZ* (gray, green) driven by a Ttk-bound region in the intron of the *jumu* gene and *Mef2* (magenta), a marker of mesoderm and muscle derivatives, in wild type (**C,C**',**E,E**',**G,G**') and $ttk_{D2\text{-}50}$ homozygous mutant (**D,D**',**F,F**',**H,H**') embryos. At stage 6, *jumu-lacZ* is transiently active in the mesoderm (arrow) of both wild type (**C,C**') and *ttk* mutant (**D,D**') embryos. At stage 14*, jumu-lacZ* is derepressed in multiple cell types of *ttk*deficient
embryos, including the ventral (**F,F**') and dorsal (**H,H**') ectoderm (arrowheads). (**C** and **D**) are ventrolateral views, (**E** and **F**) are ventral views, and (**G** and **H**) dorsal views. All embryos are orientated with anterior to the left.

Figure S10. Expression of *CG4238* in wild type and *ttk* mutant embryos
A probe against *CG4238* was used to visualize its embryonic expression pattern (gray, green), in combination with a general mesodermal marker *Mef2* (magenta). (**A-B')** In both

wild type and *ttk* mutant embryos at stage 8, *CG4238* is expressed only in the germ cells (arrowheads). (**C**-**D**') At stage 11, *CG4238* continues to be expressed in germ cells and in *ttk* mutant embryos, it starts to be expressed in the salivary gland as well (arrowheads). (**E**-**F**') At stage 13, *CG4238* is strongly derepressed in the gut (arrowheads) in *ttk* mutant embryos. (**G**-**H**') At stage 15, *CG4238* is derepressed in a subset of somatic mesoderm (arrows) and ectoderm (arrowheads) in *ttk* mutant embryos. In wild type embryos, *CG4238* continues to be expressed only in the germ cells. All embryos are lateral views, oriented with anterior to the left and dorsal to the top with the exception of embryos in **E** and **F,** which are shown in dorsolateral views.

Figure S1

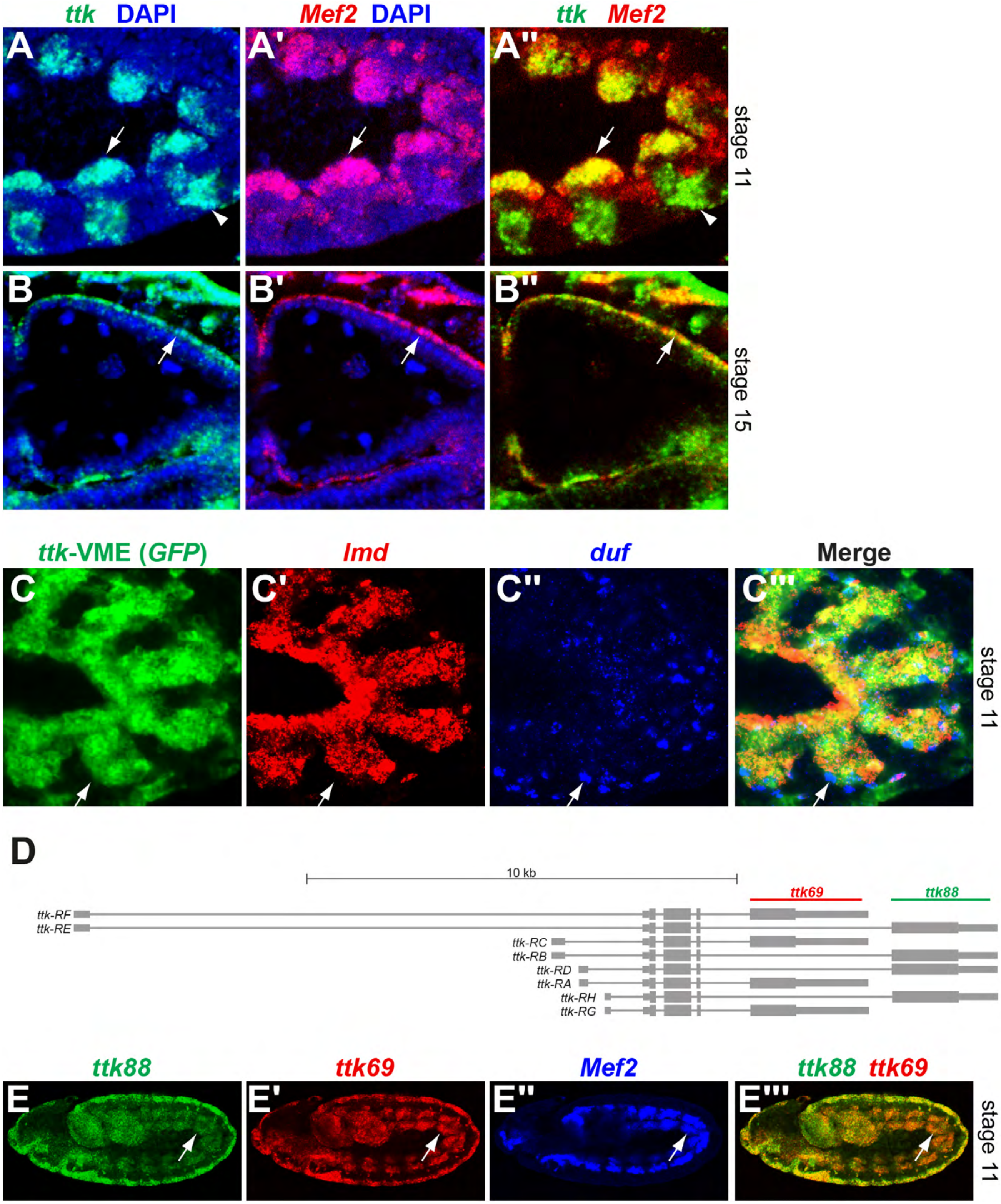
ttk DAPI
Mef2 DAPI
ttk Mef2
A
A'
A''
stage 11
B
B'
B''
stage 15
ttk-VME (GFP)
lmd
duf
Merge
C
C'
C''
C'''
stage 11
D
10 kb
ttk69
ttk88
ttk-RF
ttk-RE
ttk-RC
ttk-RB
ttk-RD
ttk-RA
ttk-RH
ttk-RG
ttk88
ttk69
Mef2
ttk88 ttk69
E
E'
E''
E'''
stage 11

Figure S2

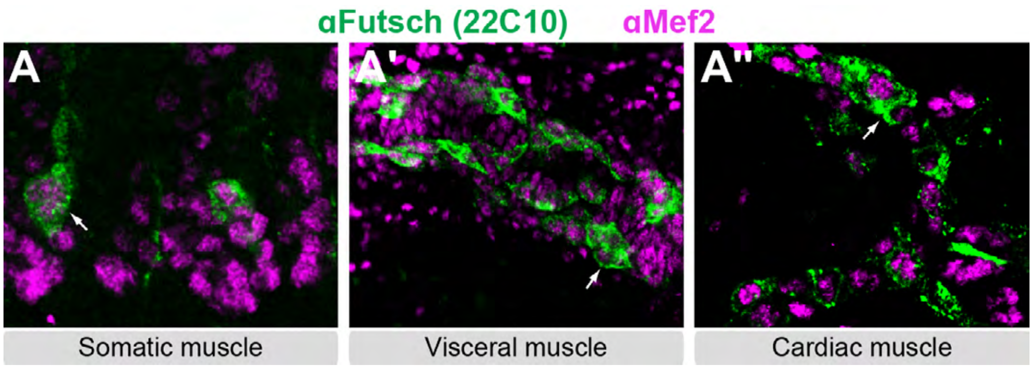

Figure S3

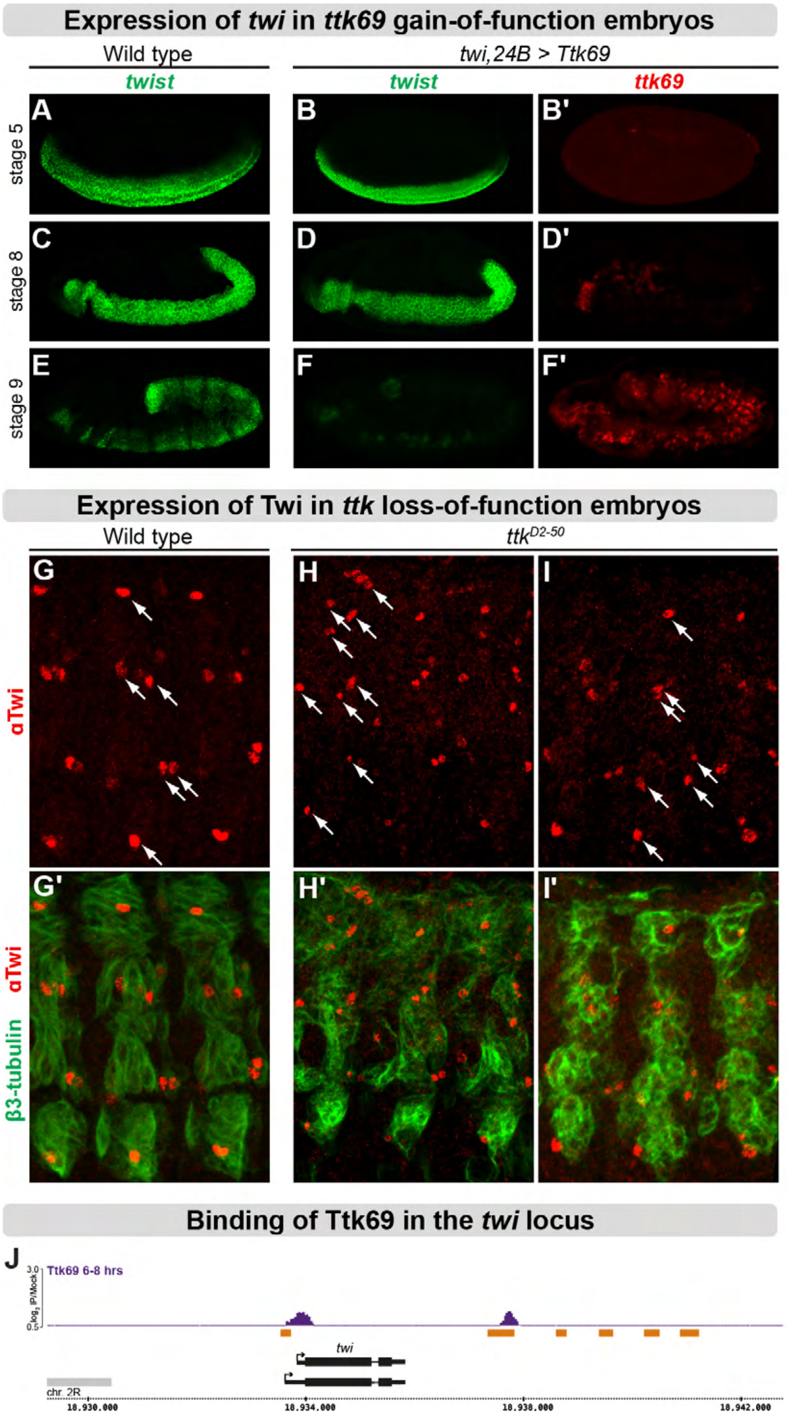

Figure S4

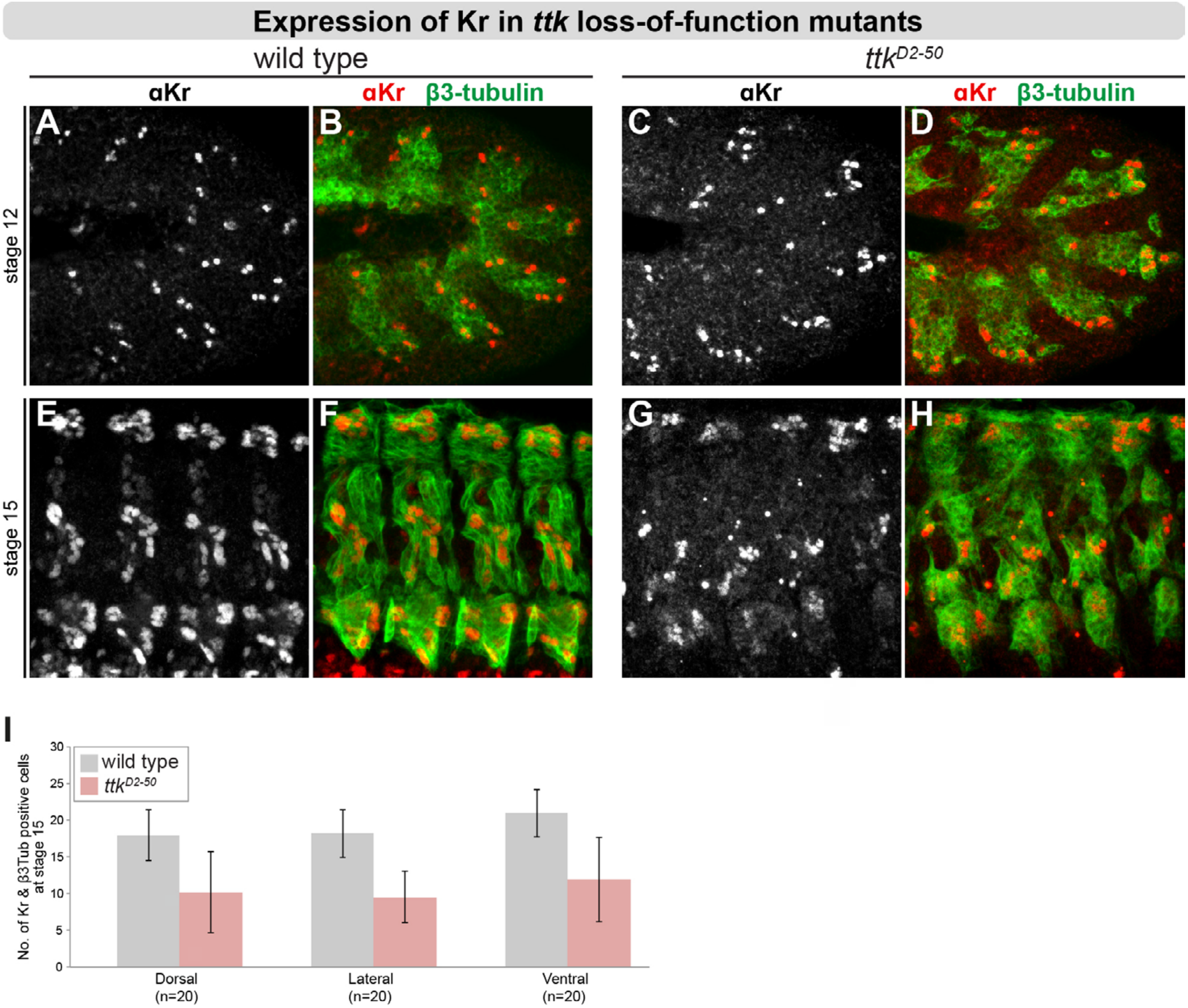

Expression of Kr in ttk loss-of-function mutants
wild type
ttkD2-50
αKr
αKr β3-tubulin
αKr
αKr β3-tubulin
A
B
C
D
stage 12
E
F
G
H
stage 15
I
No. of Kr & β3Tub positive cells at stage 15
wild type
ttkD2-50
30
25
20
15
10
5
0
Dorsal (n=20)
Lateral (n=20)
Ventral (n=20)

Figure S5

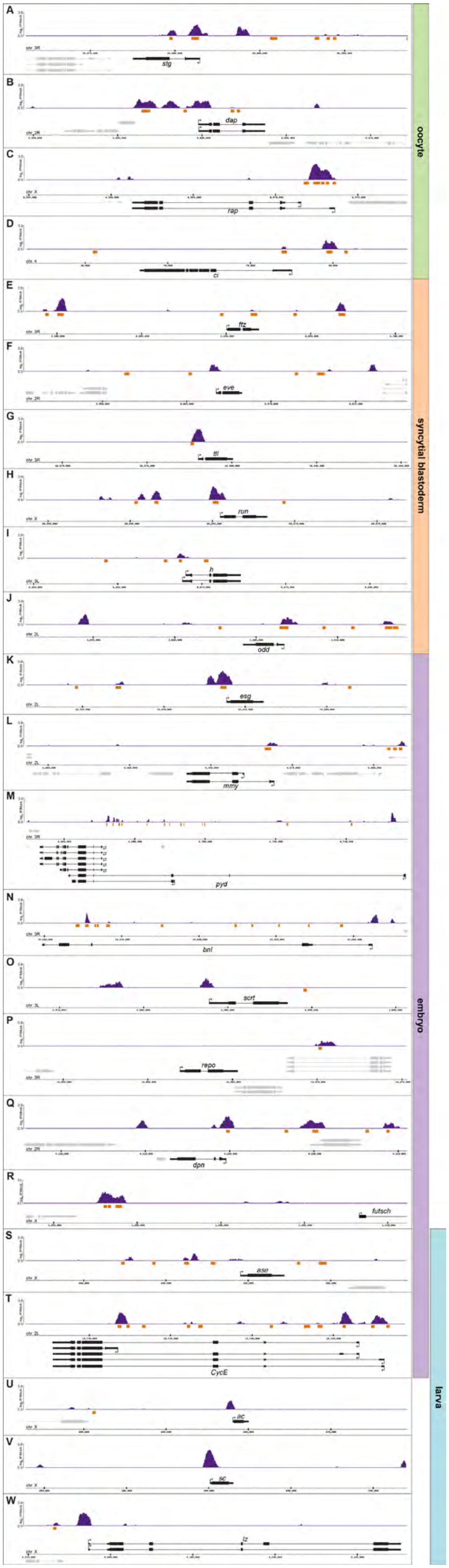
A
stg
B
dap
C
rap
D
ci
E
ftz
F
eve
G
tll
H
run
I
h
J
odd
K
esg
L
mmy
M
pyd
N
bnl
O
scrt
P
repo
Q
dpn
R
futsch
S
ase
T
CycE
U
ac
V
sc
W
lz
oocyte
syncytial blastoderm
embryo
larva

Figure S6

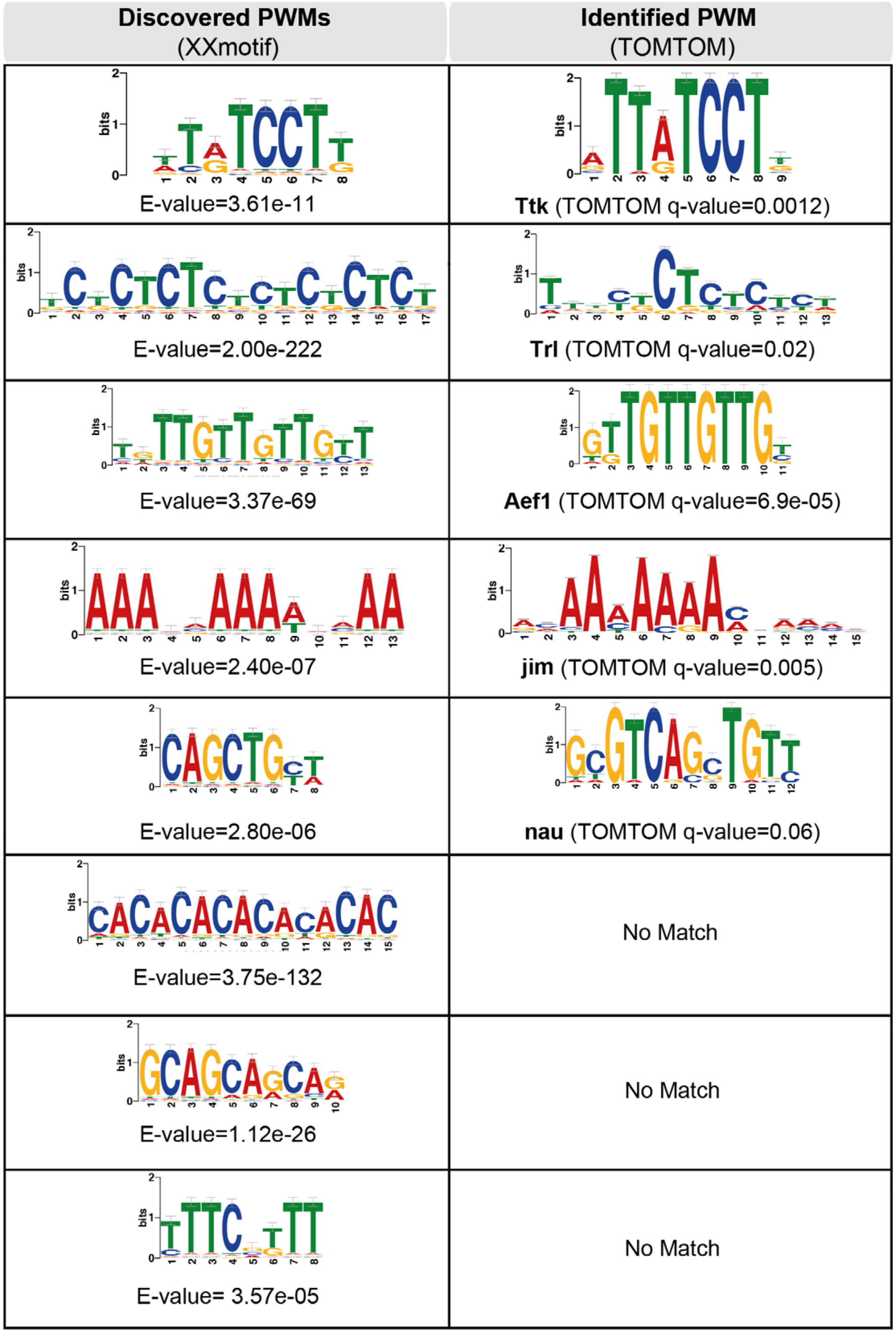
Discovered PWMs
(XXmotif)
Identified PWM
(TOMTOM)
E-value=3.61e-11
Ttk (TOMTOM q-value=0.0012)
E-value=2.00e-222
Trl (TOMTOM q-value=0.02)
E-value=3.37e-69
Aef1 (TOMTOM q-value=6.9e-05)
E-value=2.40e-07
jim (TOMTOM q-value=0.005)
E-value=2.80e-06
nau (TOMTOM q-value=0.06)
E-value=3.75e-132
No Match
E-value=1.12e-26
No Match
E-value= 3.57e-05
No Match

Figure S7

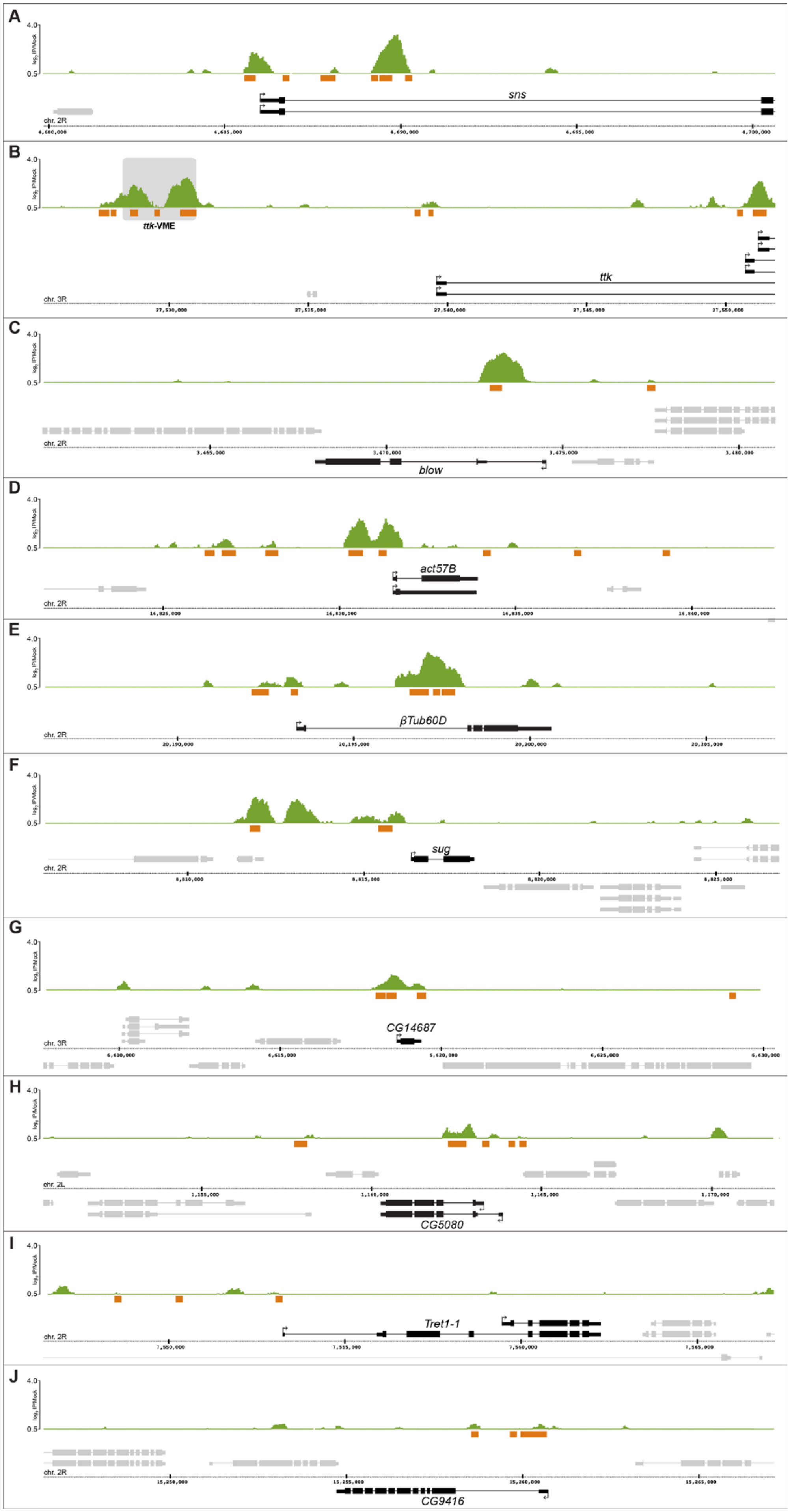
A
sns
chr. 2R
B
ttk-VME
ttk
chr. 3R
C
blow
chr. 2R
D
act57B
chr. 2R
E
βTub60D
chr. 2R
F
sug
chr. 2R
G
CG14687
chr. 3R
H
chr. 2L
CG5080
I
Tret1-1
chr. 2R
J
chr. 2R
CG9416

Figure S8

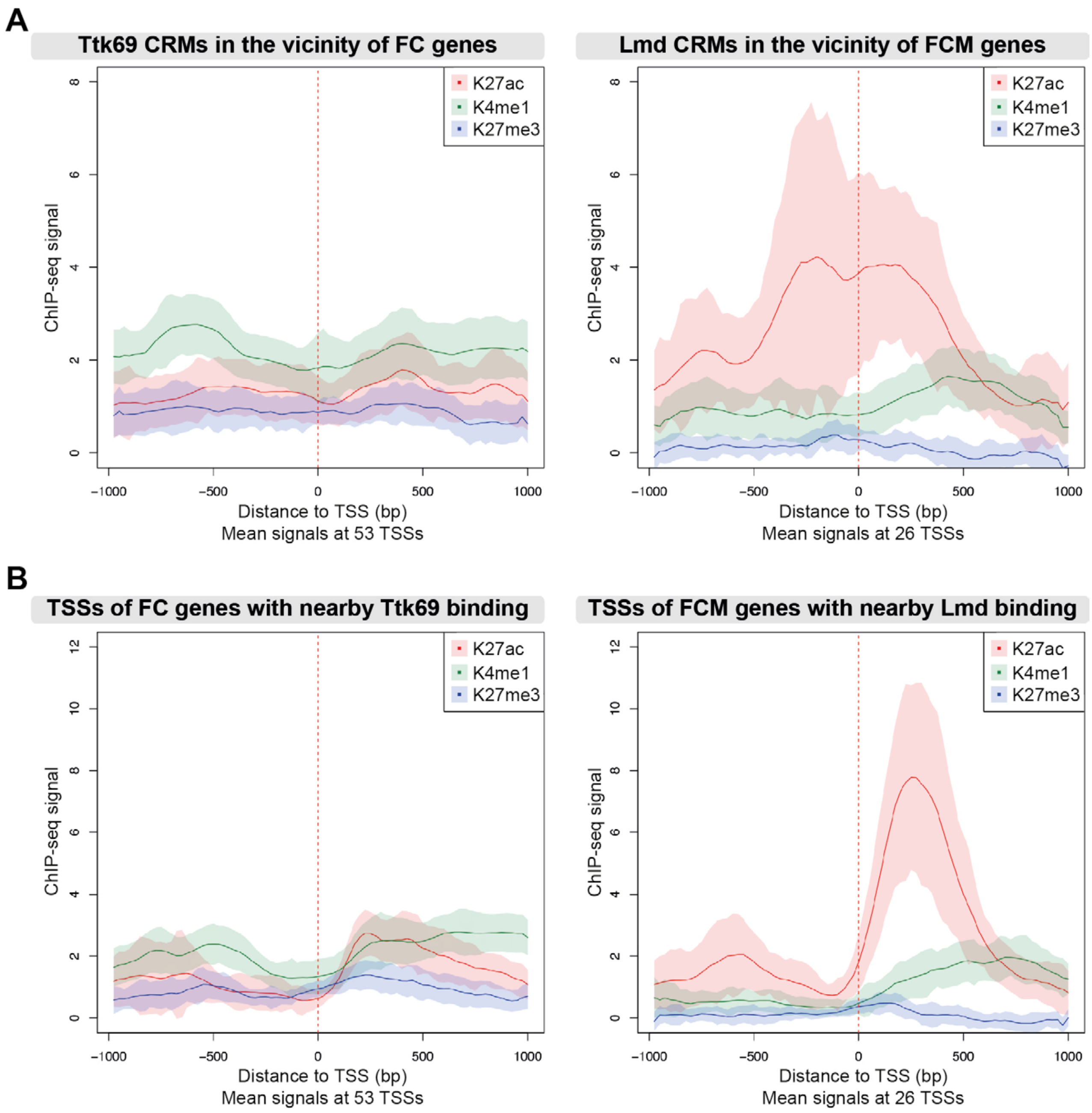
A
Ttk69 CRMs in the vicinity of FC genes
Lmd CRMs in the vicinity of FCM genes
K27ac
K4me1
K27me3
ChIP-seq signal
Distance to TSS (bp)
Mean signals at 53 TSSs
Mean signals at 26 TSSs
B
TSSs of FC genes with nearby Ttk69 binding
TSSs of FCM genes with nearby Lmd binding

Figure S9

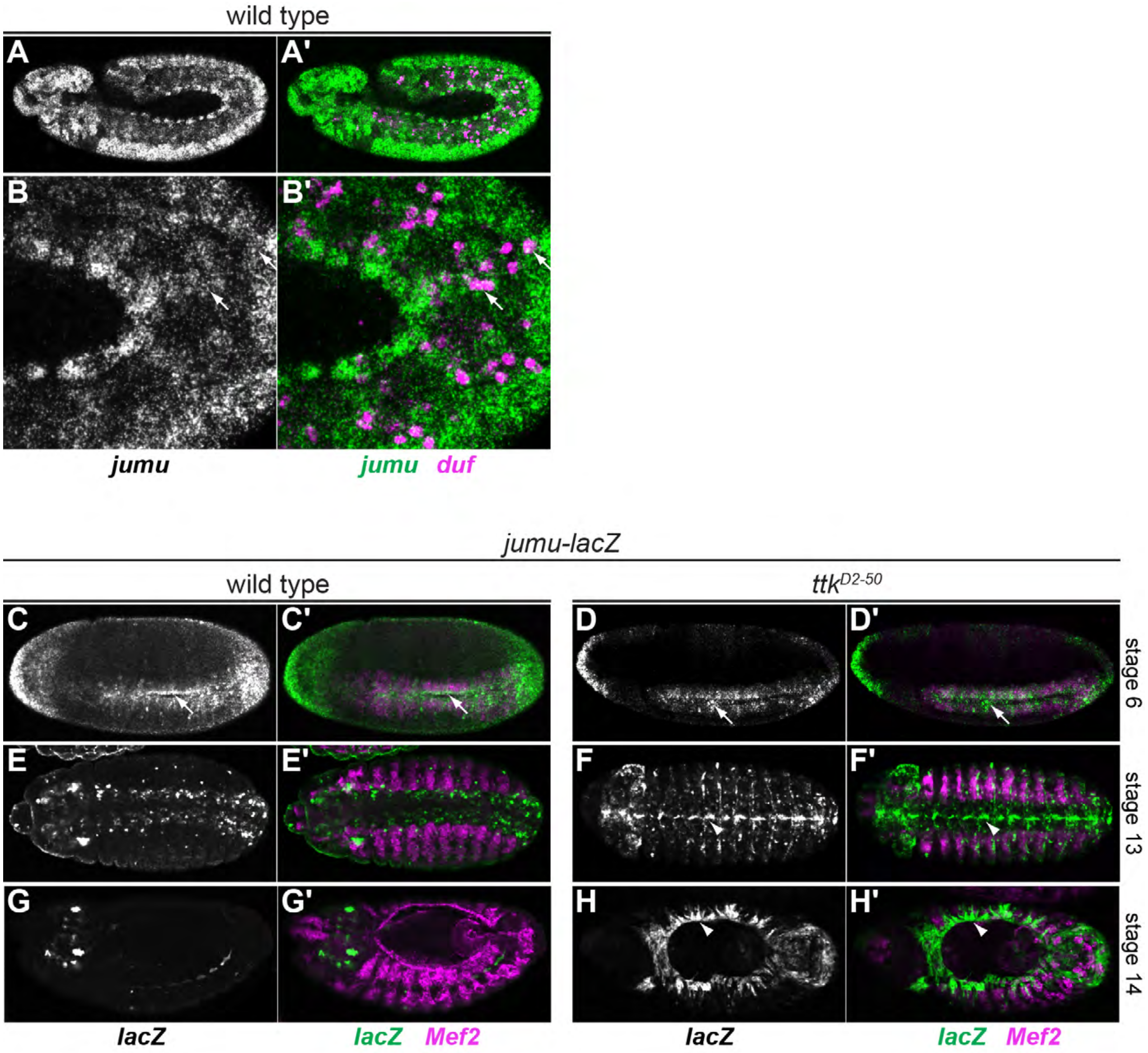
wild type
A
A'
B
B'
jumu
jumu duf
jumu-lacZ
wild type
ttk^{D2-50}
C
C'
D
D'
stage 6
E
E'
F
F'
stage 13
G
G'
H
H'
stage 14
lacZ
lacZ Mef2
lacZ
lacZ Mef2

Figure S10

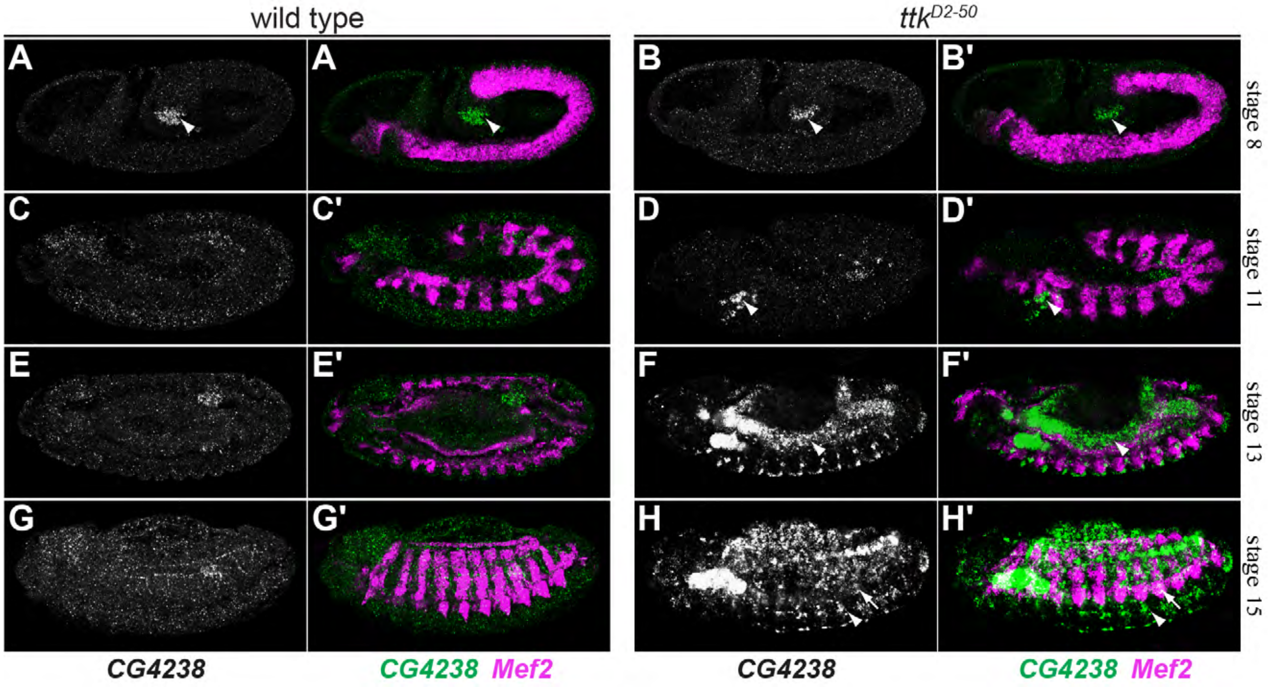

wild type
ttk^D2-50
A
A
B
B'
stage 8
C
C'
D
D'
stage 11
E
E'
F
F'
stage 13
G
G'
H
H'
stage 15
CG4238
CG4238 Mef2
CG4238
CG4238 Mef2

Download Table S1

Download Table S2

Download Table S3

Table S4: Genes with Ttk69 and Lmd co-bound CRM(s) in their vicinity

Expressed in mesoderm	Expressed outside of mesoderm	Ubiquitously expressed
FBgn0000014	FBgn0000363	FBgn0000157
FBgn0000044	FBgn0000459	FBgn0000448
FBgn0000492	FBgn0001168	FBgn0000568
FBgn0000721	FBgn0001257	FBgn0000591
FBgn0001186	FBgn0003388	FBgn0001139
FBgn0001235	FBgn0003396	FBgn0002633
FBgn0002733	FBgn0003460	FBgn0002945
FBgn0002734	FBgn0003513	FBgn0003200
FBgn0002735	FBgn0003896	FBgn0003261
FBgn0003285	FBgn0004102	FBgn0003345
FBgn0003432	FBgn0004666	FBgn0004198
FBgn0003448	FBgn0010611	FBgn0004859
FBgn0003502	FBgn0011766	FBgn0005630
FBgn0003721	FBgn0026058	FBgn0010323
FBgn0003862	FBgn0028425	FBgn0015024
FBgn0004368	FBgn0052767	FBgn0015778
FBgn0005511	FBgn0030628	FBgn0016977
FBgn0005771	FBgn0030970	FBgn0020307
FBgn0010100	FBgn0033075	FBgn0020386
FBgn0010300	FBgn0033939	FBgn0024308
FBgn0010470	FBgn0036494	FBgn0026533
FBgn0011286	FBgn0036732	FBgn0027108
FBgn0011656	FBgn0039905	FBgn0028953
FBgn0011763	FBgn0040318	FBgn0031098
FBgn0013263	FBgn0052711	FBgn0031376
FBgn0014011	FBgn0052772	FBgn0031395
FBgn0015371		FBgn0033636
FBgn0015513		FBgn0034476
FBgn0016694		FBgn0035397
FBgn0017397		FBgn0037293
FBgn0020257		
FBgn0020513		
FBgn0024244		
FBgn0024555		
FBgn0029003		
FBgn0029903		
FBgn0029927		
FBgn0029939		
FBgn0029970		
FBgn0031516		
FBgn0031895		
FBgn0032078		
FBgn0032815		
FBgn0034087		
FBgn0036030		
FBgn0036449		
FBgn0036555		
FBgn0036921		
FBgn0036945		
FBgn0037471		

FBgn0037635
FBgn0037960
FBgn0040108
FBgn0042650
FBgn0050115
FBgn0052813

Table S5: Binding of Ttk69 and Lmd in the vicinity of genes expressed in FCs, FCMs or both cell types

"+" or "-" meso indicates overlap with a CRM bound by 5 key mesodermal TFs (Zinzen et al., 2009)

Genes with Ttk69-only bound CRM(s) within 1.5kB from TSS						Genes with Lmd-only bound CRM(s) within 1.5kB from TSS					
FC+meso	FC-meso	FCM+meso	FCM-meso	FC&FCM+meso	FC&FCM-meso	FC+meso	FC-meso	FCM+meso	FCM-meso	FC&FCM+meso	FC&FCM-meso
FBgn0000097	FBgn0000180	FBgn0029082	FBgn0029504	FBgn0004907	FBgn0040395	FBgn0003285	FBgn0003175	FBgn0001142	FBgn0003076	FBgn0003285	FBgn0023023
FBgn0000099	FBgn0000606			FBgn0041145	FBgn0011656	FBgn0011571	FBgn0034657	FBgn0004133	FBgn0004919	FBgn0022959	FBgn0023081
FBgn0001180	FBgn0008651			FBgn0000472		FBgn0011592	FBgn0035036	FBgn0005427	FBgn0020766	FBgn0030061	FBgn0037843
FBgn0001316	FBgn0010341					FBgn0011817	FBgn0037066	FBgn0008649	FBgn0029148	FBgn0030771	
FBgn0001319	FBgn0025641					FBgn0020299	FBgn0039187	FBgn0010812	FBgn0035464	FBgn0033917	
FBgn0001325	FBgn0036494					FBgn0034802		FBgn0017565	FBgn0036663		
FBgn0001981	FBgn0036565					FBgn0038071		FBgn0022960	FBgn0036762		
FBgn0002561						FBgn0041096		FBgn0024238	FBgn0038369		
FBgn0003124						FBgn0050122		FBgn0024556	FBgn0038381		
FBgn0003254								FBgn0028980	FBgn0052066		
FBgn0003300								FBgn0033310			
FBgn0003430								FBgn0033782			
FBgn0003862								FBgn0034089			
FBgn0004177								FBgn0037439			
FBgn0004373								FBgn0037835			
FBgn0004449								FBgn0037848			
FBgn0004569											
FBgn0004647											
FBgn0005630											
FBgn0010105											
FBgn0010452											
FBgn0011278											
FBgn0011674											
FBgn0013725											
FBgn0014143											
FBgn0015396											
FBgn0015805											
FBgn0015907											
FBgn0017581											
FBgn0020647											
FBgn0020912											
FBgn0023526											
FBgn0026084											
FBgn0026319											
FBgn0027949											
FBgn0028369											
FBgn0028978											
FBgn0033681											
FBgn0034224											
FBgn0035128											
FBgn0036899											
FBgn0037207											
FBgn0037760											
FBgn0039350											
FBgn0051536											
FBgn0052560											
